# Transcriptional and Translational Relationship in Environmental Stress: RNAseq and ITRAQ Proteomic Analysis Between Sexually Reproducing and Parthenogenetic Females in *Moina micrura*

**DOI:** 10.3389/fphys.2018.00812

**Published:** 2018-07-02

**Authors:** Jingyi Jia, Xiangjiang Liu, Lu Li, Chengqiang Lei, Ying Dong, Guoqiang Wu, Guangfu Hu

**Affiliations:** College of Fisheries, Huazhong Agricultural University, Wuhan, China

**Keywords:** *Moina micrura*, sexual female, parthenogenetic female, RNA-Seq, iTRAQ

## Abstract

*Moina micrura* is a kind of small-bodied water flea within the family Moinidae. Similar to *Daphnia*, *M. micrura* could also switch its reproduction mode from parthenogenetic female (PF) to sexual female (SF) to adapt to the external environment. To uncover the mechanisms of reproductive switching in *M. micrura*, we used both RNA-Seq and iTRAQ analyses to investigate the differentially expressed genes (DEGs) and their protein products between SF and PF in *M. micrura*. A total of 1665 DEGs (702 up-regulated, 963 down-regulated) and 600 differentially expressed proteins (DEPs) (102 up-regulated, 498 down-regulated) were detected in SF. Correlation analyses indicated that 31 genes were expressed significantly differentially at both transcriptomic and proteomic levels, including 15 up-regulated genes and 16 down-regulated genes in SF. Meanwhile, our data also showed that 528 DEPs have discordant expression at transcript level, implying post-transcriptional (including translational) regulation. These top up-regulated genes and their protein products in SF were mainly grouped into the globin-related family, vitellogenin-related family, cuticle-related family, Hsp-related family and methyltransferases-related family, which were all involved in the reproductive switching in *Daphnia*. In contrast, a cluster of orthologous groups revealed that up-regulated genes and their protein products in PF were strongly associated with the metabolic process, which may be responsible for rapid population proliferation in *M. micrura*.

## Introduction

*Moina micrura* is a kind of small-bodied crustaceans within the family Moinidae. It is referred to as water flea but smaller than its more well-known cousins: the larger *Daphnia magna* and the medium-sized *Daphnia pulex*. *M. micrura* is the very ideal live bait for fish larvae and economically important crustaceans, and could serve as an indicator for evaluating water quality in the aquatic ecosystem (Arauzo and Valladolid, 2003). Similar to other water fleas, *M. micrura* utilizes a special reproductive strategy known as cyclical parthenogenesis, in which parthenogenesis (asexual reproduction) and sexual reproduction can be switched when the environmental characteristics change (Hobaek and Larsson, 1990; Rojas et al., 2001). When the environmental conditions are ideal and appropriate, parthenogenesis is occurred to produce a large number of parthenogenetic embryos. When facing deteriorated environmental conditions (e.g., shortened day length, low or high temperature, lack of nutrients and overpopulation), *M. micrura* could undergo an unusual transition from asexual to sexual reproduction and produce resting eggs to live through the difficult days. This reproductive strategy is an adaptation to changes in external environment; parthenogenesis contributes to the rapid proliferation in the favorable seasons, while sexual reproduction results in the increase of the genetic diversity and survival through poor environmental conditions (Barton and Charlesworth, 1998; Toyota et al., 2016). Therefore, *M. micrura* is also a great model organism for elucidating the molecular mechanism of reproductive switching in cladocerans. However, little is known about the precise mechanism of reproductive switching in *M. micrura*.

Previous studies have demonstrated that the reproductive switching of cladocerans was closely related with the changes of environmental factors including the photoperiod, temperature, food, and population density. For example, excessive temperature could induce the emergence of male and produce a large number of resting eggs (Green, 1965; Gordo et al., 1994). Short light period could significantly increase the proportion of SF and males (Baer and Owens, 1999; Toyota et al., 2015). Food is another effective factor in the sex determination of cladocerans, and its quantity and quality have a direct effect on the reproductive switching of cladocerans (Arnold, 1971). Population density also plays an important role in the reproductive switching of cladocerans. The amount of resting eggs usually shows a positive correlation with population density (Banta and Brown, 1929). In natural waters, male and SF are usually found when the population density of cladocerans is high (Carvalho and Hughes, 1983). Besides these ecological factors, several key genes have also been detected to play a pivotal role in the regulation of reproductive switching of cladocerans. For example, Kato et al. isolated five important genes involved in reproductive switching in *D. magna*, such as DM-domain (Kato et al., 2008), transformer (Kato et al., 2010), and doublesex (Kato et al., 2011). In addition, basing on the EST sequence analysis, other reproductive-specific genes were also detected, such as *doublesex/mab-3 domain*, *ubulin alpha chain*, *induction gene (CSP)* and *heat shock protein (HSP)* gene (Xu et al., 2009). These genes, which are closely related to perceiving the changes of environment, may play a key regulatory role in parthenogenesis of cladocerans. However, the reproductive switching is a complicated process in cladocerans. Little is known about which genes regulate reproductive switching, and what the interactions between transcriptional and translational control of gene expression are.

High-throughput sequencing technologies empower us to analyze alterations of an organism’s global molecular profile in response to environmental stresses. RNA sequencing (RNA-Seq) is a transcript quantification technique and has been widely and successfully applied to gene annotation, expression analysis and transcript profiling (Yang et al., 2011). As well, proteomics is a high-throughput approach to address gene functions that cannot be offered by genome sequences and is the most direct way to confirm the function of a gene (Ahsan et al., 2008). Isobaric tags for relative and absolute quantitation (iTRAQ) is the second generation of gel-free proteomic analysis that allows for a more accurate quantitation of proteins than low-throughput proteomic methods, such as traditional two-dimensional gel-based approaches. Recently, transcriptome analysis has been used to determine the DEGs between PF and SF in *D. pulex* (Toyota et al., 2015) and *D. similoides* (Zhang et al., 2016), respectively. However, as far as we know, there is no study to conduct proteomic profiling to examine the proteins involved in the reproductive switching under environmental stresses.

The aim of this study is to further investigate the key genes and their protein products involved in reproductive switching. To achieve this, using *M. micrura* as model, both RNA-Seq and iTRAQ technology are used to identify the differentially expressed mRNAs and proteins between SF and PF. These findings will provide important information on the molecular mechanism of the reproductive switching in *M. micrura*.

## Materials and Methods

### Sample Preparation

**FIGURE 1:**
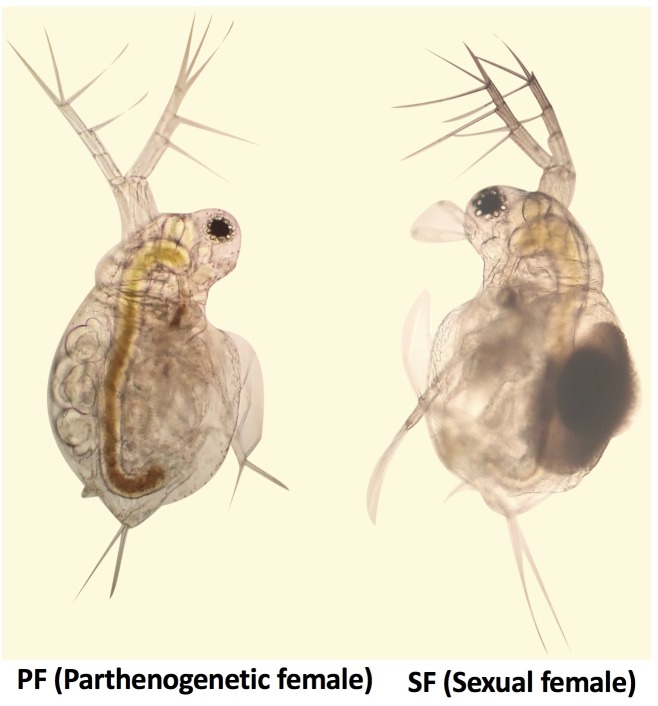
The photograph of *Moina micrura* female.

*Moina micrura* was isolated from the South Lake (Wuhan city, China), and the pure lines were established by monoclonal culture in our laboratory. Healthy parthenogenetic organisms were cultivated under a 12 h light/12 h dark photoperiod at 25°C, and fed chlorella for 1 year. According to the biological characteristics of *M. micrura*, reproduction switching occurs when the population density reaches a certain level. Healthy SF and PF were collected and their reproductive state was confirmed, respectively, using OLYMPUS BA200 microscope (**Figure 1**), and put into cryogenic vials (100 per vial). Then samples in the cryogenic vials were washed three times with sterilized water. After that, water was drained and the samples were immediately frozen in liquid nitrogen and stored at -80°C until further processing. In this study, three tubes of SF and PF were collected for transcriptomic and proteomic sequencing, respectively.

### Total RNA Isolation and cDNA Library Construction

Total RNA was extracted from SF and PF using TRIzol reagent (Invitrogen, United States) according to the manufacturer’s instructions, and genomic DNA was removed using DNase I (TaKara). The Nanodrop 2000 spectrophotometer was used to assess sample purity and RNA concentration, and the quality of RNA was analyzed on an Agilent 2100 Bioanalyzer using the RNA 6000 Nano kit (Agilent Technologies, Santa Clara, CA, United States). All samples displayed a 260/280 ratio greater than 2.0 and RNA integrity numbers (RIN) ≥7.5. Before the construction of library, ribosomal and viral RNA were removed and poly(A)+ mRNA was isolated with magnetic Oligo-dT beads (Invitrogen, United States), and then 10 μg of total RNA for each sample was used for the construction of libraries by using a TruSeq^TM^ RNA sample preparation Kit from Illumina (San Diego, CA, United States), all performed according to the Illumina protocol. Thereafter, libraries were sequenced using Illumina HiSeq^TM^ 2000 system at Majorbio Biotech Co., Ltd. (Shanghai, China). We chose a read depth of 600 million 150-bp single end reads. All raw-sequence read data were deposited in NCBI Sequence Read Archive (SRA^1^) with accession number SRP136759.

### Transcriptome *de Novo* Assembly and Bioinformatics Analysis

The raw paired end reads were trimmed and quality controlled by SeqPrep^2^ and Sickle^3^ with default parameters. Then clean data from the samples (SF and PF) were used to do *de novo* assembly with Trinity^4^ and all the assembled full-length sequences were named unigenes (Grabherr et al., 2011). All the unigenes were predicted and used for Blastx search and annotation against the NR, Swiss-Prot, COG, and Kyoto Encyclopedia of Genes and Genomes (KEGG) databases (Camacho et al., 2009). Functional annotation by gene ontology (GO) terms was analyzed by using Blast2GO software (Conesa et al., 2005), and GO functional classification for unigenes was analyzed by using WEGO software (Ye et al., 2006). The read counts were further normalized into FPKM values. The fold changes were calculated by using RSEM software v 1.2.7 (Li and Dewey, 2011), and the DEGs were analyzed using the R Bioconductor package, edgeR (Robinson et al., 2010). The *P*-value set the threshold for the differential gene expression test. The threshold of the *P*-value in multiple tests was determined by the value for the false discovery rate (FDR) (Benjamini and Yekutieli, 2001). DEGs were screened with a cut-off conditions of fold change (FC) > 2.0 and FDR ≤ 0.001.

### Protein Preparation and iTRAQ Labeling

The *M. micrura* samples were sonicated in ice with appropriate amount of lysis buffer (8 M urea, 0.3% SDS) and protease inhibitors (Thermo, United States). The proteins were reduced with 10 mM DTT (final concentration) at 56°C for 1 h and alkylated by 55 mM IAM (final concentration) in the darkroom for 1 h. The reduced and alkylated protein mixtures were precipitated by adding 4× volume of chilled acetone at -20°C overnight. After centrifugation at 4°C, 30,000 *g*, the pellet was dissolved in 0.5 M TEAB (Applied Biosystems, Milan, Italy) and sonicated in ice. After centrifugation at 30,000 *g* at 4°C, an aliquot of the supernatant was taken to determine the protein concentration with a 2-D Quant Kit (GE Healthcare). The proteins in the supernatant were kept at -80°C for further analysis. More details for iTRAQ labeling are provided in the Supplementary Supporting Information.

### Liquid Chromatography Tandem Mass Spectrometry (LC/LC–MS/MS) Analysis

Each fraction was resuspended with loading buffer [5 mM ammonium formate containing 2% acetonitrile (ACN); pH = 10] and separated by high-pH reversed-phase liquid chromatography (RPLC, Acquity Ultra Performance LC; Waters, Milford, MA, United States). The solvent A and solvent B was 2% ACN (pH = 10, adjusted by ammonia) and 80% ACN (pH = 10, adjusted by ammonia), respectively. The gradient elution was performed with 0–30% solvent B for 2–38 min and 30–100% solvent B for 38–40 min on a high-pH RPLC column (C18, 1.7 μm, 2.1 mm × 150 mm; Waters, United States). More details of LC/LC–MS/MS analysis are provided in the Supporting Information. All mass spectrometry proteomics data were deposited in Integrated Proteome Resources (iProX^5^) with accession number IPX0001186000.

### Analysis of the Identified Proteins

The raw data obtained from LC/LC–MS/MS analysis were processed using Proteome Discoverer^TM^ Software 2.1 (Thermo, United States). The search parameters included uniprot- organism6669-*D. pulex* as protein database, Iodoacetamide as Cys alkylation, oxidation (M), acetyl (Protein N-Terminus) and iTRAQ8plex (Y), as the potential dynamic modifications, and iTRAQ8plex (K), iTRAQ8plex (N-Terminus), as the static modifications. Functional annotations of the proteins were conducted with the Blast2GO (Gene Ontology) program against the non-redundant protein database (NR; NCBI), and the differentially annotated proteins were further assigned to KEGG database. The significant *p*-value of the difference between the samples was calculated by using the *t*-test function in the R language. For identifying significantly up- or down-regulated proteins, the threshold values of SF/PF or PF/SF ratios were ≥1.50 or ≤0.67 (≥1.5-fold) and the *p*-value < 0.05 in both two iTRAQ analyses.

### Correlation Analysis Between Transcriptome and Proteome Data

To assess the potential relevance of quantitative information between mRNA and proteins, the cut off values (DEGs: |Fold change (FC)| > 2.0 and FDR ≤ 0.001; DEPs: *p*-value < 0.05 and |FC| > 1.50) were used to screen the subsets of mRNA and proteins with apparent expression. Then, we used RNA-seq data as a searchable database, all identified protein sequences were analyzed and queried with the RNA-seq data. To further dig out the information from proteomes, the correlation analysis was conducted between DEPs and the whole transcript database.

### Real-Time Quantitative PCR (RT-qPCR) Validation

Total RNA of *M. micrura* was extracted from SF and PF samples with TRIzol reagent (Invitrogen, United States), respectively. The Nanodrop 2000 spectrophotometer was used to assess sample purity and RNA concentration, and 10 μg total RNA from PF and SF were reversely transcribed by PrimeScript RT reagent kit (Takara, Dalian, China), respectively. The RT samples obtained were subjected to qPCR using a ABI 7500 real-time PCR system (Applied Biosystems, United States) with specific primers for DEGs, respectively (see Supplementary Table S1 for primer sequences and PCR condition). In these experiments, the comparative Ct method (2^-ΔΔCt^) of relative quantification (Livak and Schmittgen, 2001) was used to analyze the real-time quantitative PCR data, using QuantStudio^TM^ Real-Time PCR Software, Version 1.2. Parallel real-time PCR for *GAPDH* was also conducted to serve as the internal control. The sizes of the amplified products were confirmed through gel electrophoresis.

### Data Analysis

All the data were subjected to statistical analysis with SPSS 19.0 software (IBM Corp, Armonk, NY, United States). Physiological data were analyzed using Student’s *t*-test and the Tukey method for one-way ANOVA at 95% confidence. Real-time RT-PCR results were evaluated with the Student’s *t*-test. The data were expressed as the means ± standard errors of the mean (SEM). Differences between experimental groups were considered as significant at *P* < 0.05.

## Results

### RNA-Seq Data and Identification of DEGs

**FIGURE 2:**
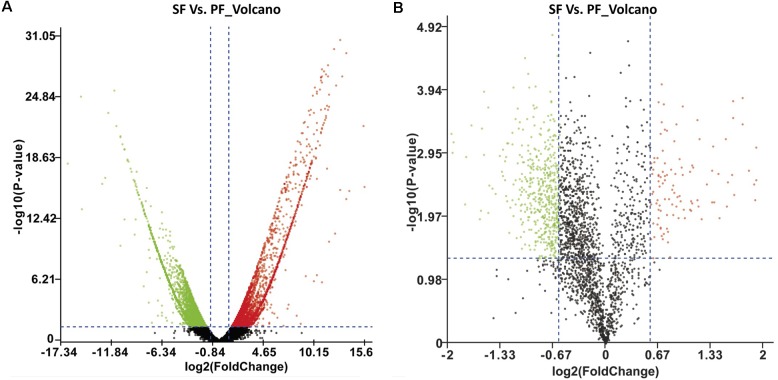
Volcano plot of DEGs and DEPs in SF and PF. **(A)** Volcano plot of DEGs in SF and PF. Splashes represent different genes, and the black splashes mean genes without significant different expression. The red splashes mean significantly up-regulated genes in SF, and the green splashes mean significantly down-regulated genes in SF. **(B)** Volcano plot of DEPs in SF and PF. Each point represents a specific protein, and the black points are the non-significant different proteins. The red dots indicate the significantly up-regulated proteins in SF, and the green dots indicate the significantly down-regulated proteins in SF. SF, sexual female; PF, parthenogenetic female, -log10 (*p*-value): the corrected *p*-value.

To survey the key genes in reproductive switching, a high-throughput transcriptome was used to compare mRNA expression profiles between SF and PF. In the present study, the cDNA libraries for SF and PF were established and sequenced. The numbers of clean reads obtained from SF and PF of *M. micrura* were 77,923,354 and 68,509,476, respectively, and the read length was 145 bp and all Q30 were higher than 96%. After quality filtering, 69,857 unigenes were acquired, in which the average length was 995 bp. For these unigenes, 20,131 (28.8%), 24,282 (34.8%), 8,807 (12.6%), 24,543 (35.1%), and 13,213 (18.9%) were annotated to NR, Swiss-prot, KEGG, COG, and GO database, respectively. Furthermore, fragments per kilobase of transcript per million fragments (FPKM) analysis showed that total 1665 unigenes (FC > 2.0 and FDR < 0.001) were differently expressed between SF and PF. Compared to PF, 963 genes were up-regulated and 702 genes were down-regulated in SF (**Figure 2A**).

### GO and KEGG Pathway Enrichment Analysis of DEGs

**FIGURE 3:**
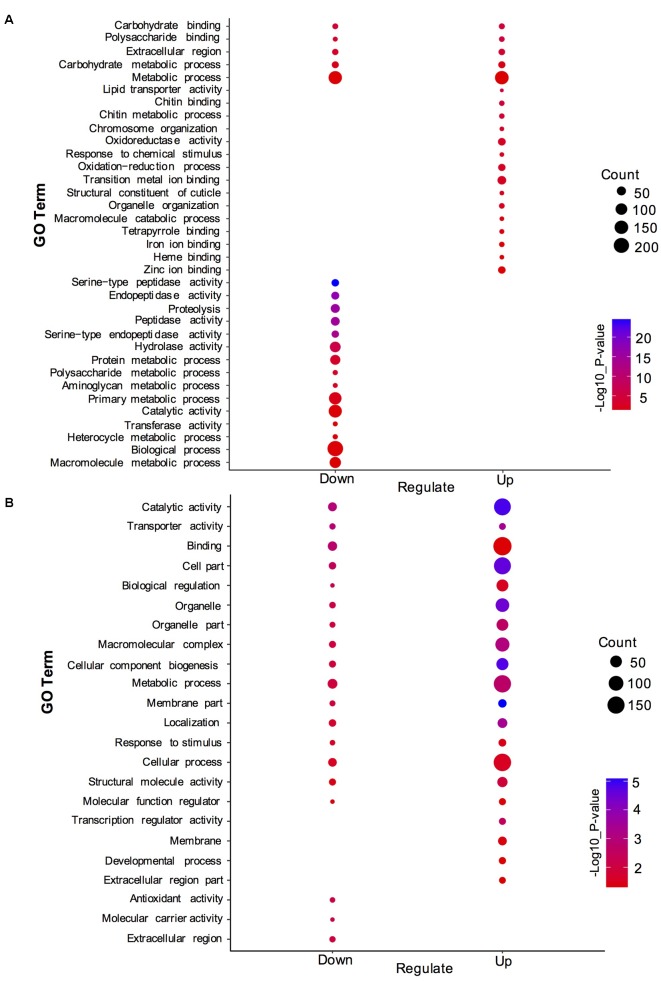
Gene ontology (GO) enrichment analysis of DEGs and DEPs in SF and PF. **(A)** Statistics of top 20 enriched GO terms for DEGs in SF and PF. **(B)** Statistics of top 20 enriched GO terms for DEPs in SF and PF. Up, up-regulated genes or proteins; Down, down-regulated gens or proteins.

**FIGURE 4:**
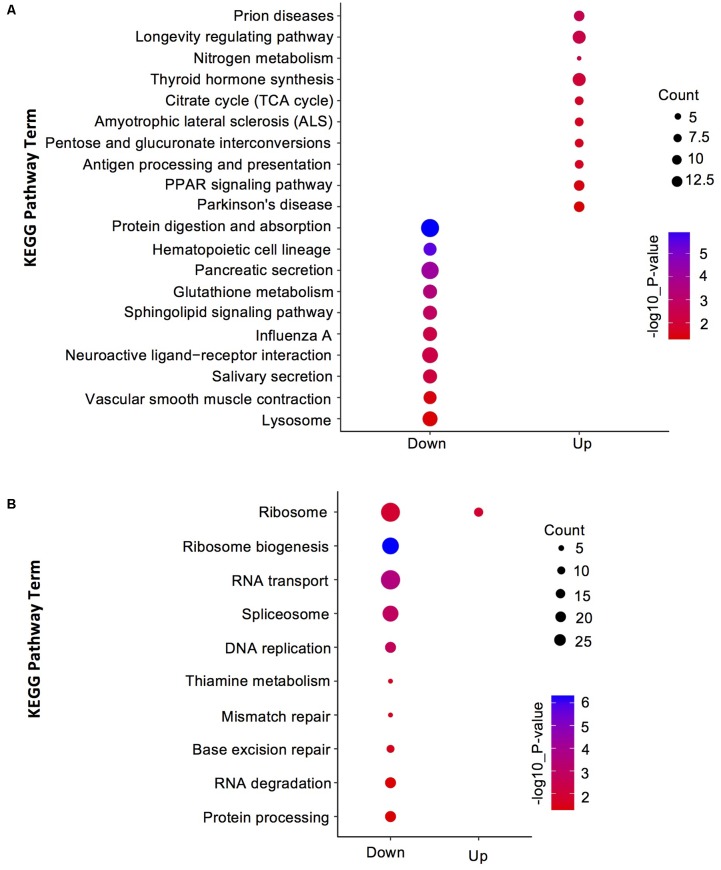
Kyoto Encyclopedia of Genes and Genomes (KEGG) pathway enrichment analysis for DEGs and DEPs in SF and PF. **(A)** Statistics of top 10 enriched pathways for DEGs in SF and PF. **(B)** Statistics of top 10 enriched pathways for DEPs in SF and PF. Up, up-regulated genes or proteins; Down, down-regulated gens or proteins.

To understand the distribution of DEGs at a macro level, the set of DEGs between the SF and PF were mapped in accordance with the GO terms and the KEGG pathway, respectively. GO enrichment analysis showed that the up-regulated genes in SF were highly related to cuticle (e.g., carbohydrate binding, polysaccharide binding, chitin binding, chitin metabolic process, and structural constituent of cuticle), hemoglobin (e.g., iron ion binding, heme binding and tetrapyrrole binding), oxidative stress (e.g., oxidoreductase activity and oxidation-reduction process), and Vg (e.g., lipid transporter activity) (**Figure 3A**). Yet, most down-regulated genes in SF were grouped in the metabolic process (e.g., macromolecule/heterocycle/aminoglycan metabolic process), primary metabolic process (e.g., protein metabolic process, proteolysis and carbohydrate/polysaccharide metabolic process), catalytic activity (e.g., hydrolase activity and transferase activity), and peptidase activity (e.g., endopeptidase activity and serine-type peptidase/endopeptidase activity) (**Figure 3A**). KEGG pathway enrichment analysis demonstrated that most up-regulated genes in SF were widely related with organismal systems (e.g., longevity regulating pathway, PPAR signaling pathway etc.) and metabolism (e.g., citrate cycle, pentose and glucuronate interconversions etc.). In addition, organismal systems (e.g., protein digestion and absorption, lysosome etc.) and environmental information processing (e.g., neuroactive ligand-receptor interaction and sphingolipid signaling pathway) were found to be the most-enriched pathways in down-regulated genes (**Figure 4A**).

### Critical DEGs Involved in Reproductive Switching in *M. micrura*

To further understand the mechanism of reproductive switching in *M. micrura*, the 1665 DEGs were further filtered, and 120 up-regulated and 103 down-regulated genes (FC ≥ 100) were identified in SF, respectively. GO analysis indicated that these 120 up-regulated genes in SF were mainly grouped into the following functions (number of genes): carbonate dehydratase activity (3), catalytic activity (2), chitin binding (2), DNA binding (9), G-protein coupled receptor activity (2), hydrolase activity (3), metal ion binding (3), molecular function (5), nucleic acid binding (2), oxidoreductase activity (3), peptidase activity (2), protein binding (17), protein kinase activity (6), RNA binding (3), serine-type endopeptidase inhibitor activity (2), structural constituent of cuticle (4), transcription factor activity (5), transferase activity (5), and zinc ion binding (6) (Supplementary Table S2). We also performed GO analysis on 103 down-regulated genes in SF, and results showed that these annotated genes were mainly grouped into the following functions (number of genes): actin binding (2), amino acid transporter activity (2), calcium activated chloride channel (2), calcium ion binding (2), carbohydrate binding (2), catalytic activity (3), cysteine-type peptidase activity (2), DNA binding (3), GTPase activity (4), hydrolase activity (2), ion channel activity (2), metal ion binding (2), molecular function (5), nucleic acid binding (3), poly(A) RNA binding (4), protein binding (12), RNA binding (3), serine-type endopeptidase activity (3), transcription factor activity (2), transferase activity (2), transmembrane transporter activity (2), transporter activity (2), and zinc ion binding (4) (Supplementary Table S3).

Among these up-regulated DEGs in SF, we further identified a few key genes that may be related to reproduction, such as *chitotriosidase-1* (*Chit1*), *probable chitinase 3* (*Cht3*), *ATP-dependent RNA helicase DDX54* (*Ddx54*), *hemoglobin*, *phosphatidylethanolamine-binding protein homolog F40A3.3* (*F40A3.3*), *Vg*, *protein-lysine N-methyltransferase Mettl10* (*Mettl10*), *alpha-crystallin A chain* (*Cryacra*), *heat shock protein Hsp-16.2* (*Hsp16.2*), *sex-lethal homolog* (*Sxl*), *endocuticle structural glycoprotein SgAbd-8* (N/A^a^), *cuticle protein 6* (N/A^b^), *cuticle protein 7* (N/A^c^), and *pupal cuticle protein 27* (*Pcp27*) (**Table 1**).

**Table 1:** Critical genes involved in reproductive switching in SF.

Gene	FC^SF^/_PF_	*P*-value	Function
*Chit1*	312.3	2.61E-14	Chitin binding
*Cht3*	602.4	4.51E-17	Chitin binding
*N/A^a^*	507.9	7.06E-23	Structural constituent of cuticle
*N/A^b^*	155.9	1.83E-11	Structural constituent of cuticle
*N/A^c^*	2034.2	4.09E-27	Structural constituent of cuticle
*Pcp27*	142.3	1.48E-11	Structural constituent of cuticle
*Ddx54*	214.0	9.54E-13	Estrogen receptor binding
*Hemoglobin*	3030.3	1.13E-24	Iron ion binding
*F40A3.3*	133.0	7.62E-11	Lipid binding
*Vg*	449.3	6.10E-22	Lipid transport
*Mettl10*	1759.1	2.19E-26	Methyltransferase activity
*Cryaa*	8091.6	8.87E-32	Protein binding
*Hsp-16.2*	1074.7	7.78E-22	Response to heat
*Sxl*	246.2	2.57E-13	Sex determination

*N/A^*a*^, endocuticle structural glycoprotein SgAbd-8; N/A^*b*^, cuticle protein 6; N/A^*c*^, cuticle protein 7.*

### Protein Identification and Quantitation

**FIGURE 5:**
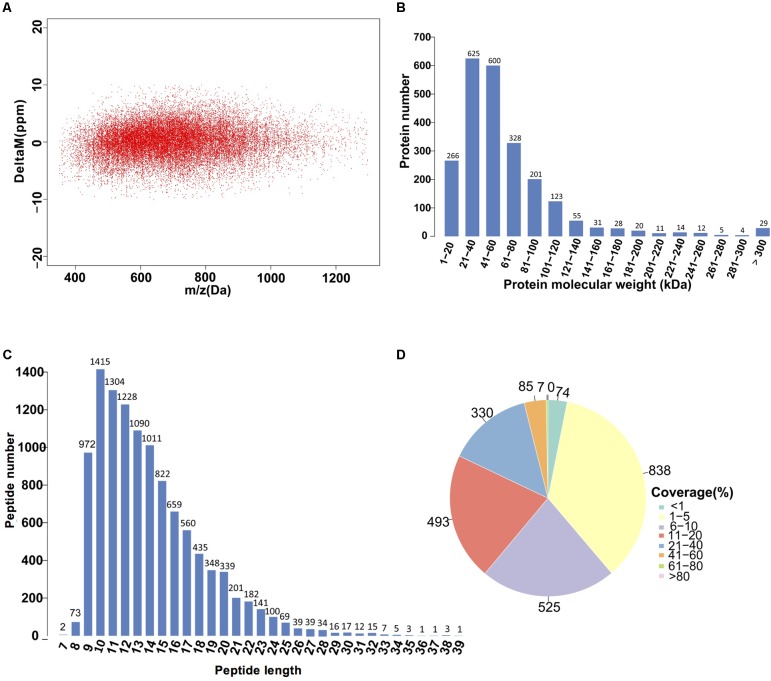
Identification and analysis of the proteome in PF and SF. **(A)** Distribution of peptide segment matching error. **(B)** The distribution of protein molecular weight. The *x*-axis shows the size of the identified protein molecular weight (unit: kilodalton, kDa), and the *y*-axis represents the number of identified proteins responsed for the corresponding size. **(C)** The distribution of peptide length. The *x*-axis indicates the length of the peptide (the number of amino acids), and the *y*-axis indicates the ratio of the peptide length to the total number of peptides. **(D)** Distribution of the protein sequence coverage. The number outside the fan indicates the coverage of the protein number in this interval.

Given the advantage of high resolution and high-quality accuracy, the Q-Exactive mass spectrometer was used to detect the DEPs between SF and PF. The iTRAQ analysis of *M. micrura* global proteome revealed 2352 protein hits in Proteome Discoverer. The error in the matching of the peptide segment found in the database was below 0.05Da (**Figure 5A**). The molecular weight of most proteins was shown in **Figure 5B**. As shown in **Figure 5C**, the peptide length was around in 9–20 aa, among which 9–15 aa interval was the peak area. The numbers of peptides identified into the proteins was exhibited in **Figure 5D**, which showed that most of protein sequence coverage was at 1–40%.

### Identification and Enrichment Analysis of DEPs

A total of 600 DEPs were identified between SF and PF (FC ≥ 1.5 and *p*-value < 0.05), among which included 102 up-regulated proteins and 498 down-regulated proteins in SF (**Figure 2B**). GO analysis showed that most DEPs were grouped into cellular component (e.g., cell part, macromolecular complex, organelle etc.), binding, cellular process, and metabolic process (**Figure 3B**). Additionally, most up-regulated DEPs in SF were preferentially related to developmental process and transcription regulator activity (**Figure 4A**). In addition to the GO analysis, KEGG pathway enrichment analysis was also used to further elucidate the putative functions of these DEPs. The results demonstrated that up-regulated proteins in SF were mainly involved in the genetic information processing, especially translation process relating to RNA transport, ribosome and ribosome biogenesis in eukaryotes, while only ribosome was found to be the most-enriched pathways in down-regulated genes in SF (**Figure 4B**).

### Critical DEPs Involved in Reproductive Switching in *M. micrura*

To further understand the mechanism of reproductive switching in *M. micrura*, 50 up-regulated (FC ≥ 1.5) and 130 down-regulated proteins (FC ≥ 1.6) were further identified in SF. Based on the GO analysis, the annotated 50 up-regulated DEPs in SF were mainly grouped into the following functions (number of proteins): calcium ion binding (2), GTPase activity (2), iron ion binding/heme binding (2), lipid transport (2), methyltransferase activity (2), oxidation-reduction process (4), oxidoreductase activity (2), protein binding (5), response to heat (2), response to oxidative stress (2), structural constituent of ribosome (5) (Supplementary Table S4). In addition, we also performed GO analysis on 130 down-regulated proteins in SF. These annotated proteins were mainly grouped into the following functions (number of proteins): calcium ion binding (4), carbohydrate metabolic process (4), catalytic activity (2), cellular component biogenesis (3), chromatin binding (2), DNA helicase activity (2), enzyme regulator activity (3), establishment of localization (6), establishment of protein localization (3), hydrolase activity (8), membrane-enclosed (2), metabolic process (3), metal ion binding (2), nucleic acid binding (10), organic cyclic compound binding (3), organic substance metabolic process (2), oxidation-reduction process (5), peptidase inhibitor activity (2), regulation of metabolic process (2), response to stimulus (4), ribonucleoprotein complex biogenesis (3), structural constituent of ribosome (11), structural molecule activity (3), transcription factor activity (2), unfolded protein binding (2), and zinc ion binding (2) (Supplementary Table S5).

Among these up-regulated DEPs in SF, we further identified a few key proteins that might be related to reproduction, including bolA-like protein (G0274169), ferritin subunit (Ferh), hemoglobin, cytoglobin-2 (Cygb2), Vg, Vg2, protein-lysine *N*-methyltransferase Mettl10 (Mettl10), NADH dehydrogenase 1 alpha subcomplex assembly factor 5 (Ndufaf5), heat shock protein (ECU02_0100), heat shock protein (Hsp-16.2), and pupal cuticle protein 20 (Pcp20) (**Table 2**).

**Table 2:** Critical proteins involved in reproductive switching in SF.

Protein	Gene	FC^SF^/_PF_	*P*-value	Function
BolA-like protein G0274169	*G0274169*	2.17	0.0019	Chitin binding
Pupal cuticle protein 20	*Pcp20*	1.60	0.0114	Structural constituent of cuticle
Ferritin subunit	*Ferh*	2.25	0.0060	Ferric iron binding
Hemoglobin	*Hemoglobin*	2.89	0.0007	Iron ion binding/heme binding
Cytoglobin-2	*Cygb2*	1.82	0.0102	Iron ion binding/heme binding
Vitellogenin	*Vg*	3.55	0.0020	Lipid transport
Vitellogenin-2	*Vg2*	3.04	0.0025	Lipid transport
Methyltransferase-like protein 10	*Mettl10*	2.28	0.0007	Methyltransferase activity
Arginine-hydroxylase NDUFAF5	*Ndufaf5*	1.87	0.0077	Methyltransferase activity
Heat shock protein Hsp-16.2	*Hsp-16.2*	2.11	0.0085	Response to heat
Heat shock protein ECU02_0100	*Ecu02_0100*	1.59	0.0025	Response to heat

### Correlation Analysis Between Transcriptome and Proteome Data in SF and PF

**FIGURE 6:**
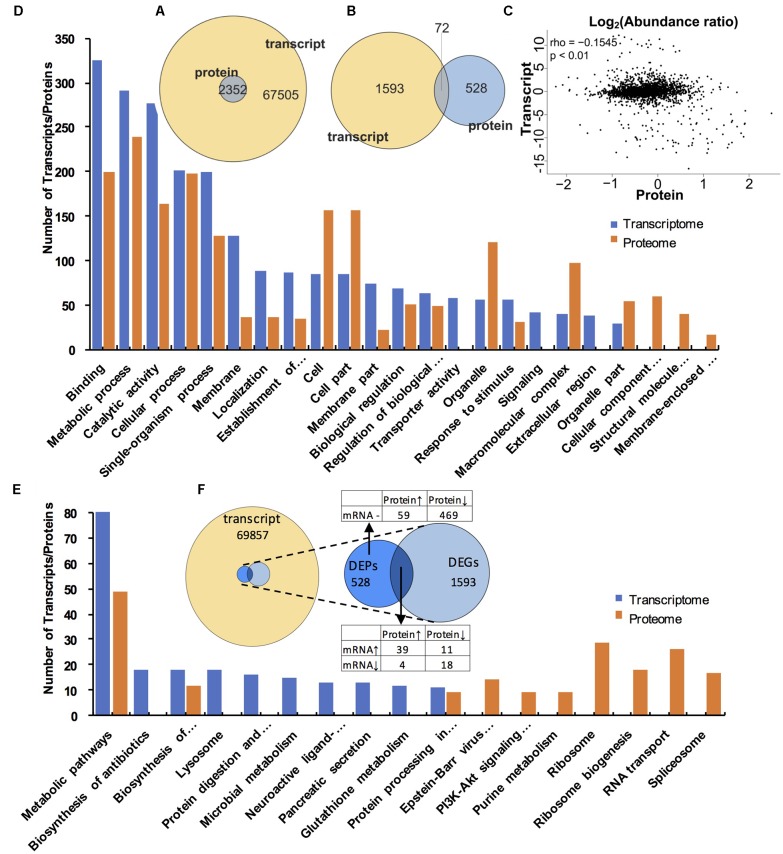
Comparative analysis of proteomic and transcriptomic data. **(A)** Venn diagrams of all transcripts and proteins. The image represents all transcripts and proteins. **(B)** Venn diagrams of DEGs and DEPs. The circle of different colors represents the different omics data of the same sample, yellow represents the transcript, and the blue represents the protein, where the values represent the common and specific transcripts and proteins in the same sample, and the sum of all the numbers within the circle represents the number of transcripts and proteins. The rounded region of the circle represents the number of DEGs and DEPs shared by the two groups. **(C)** The correlation scatter diagram of the expression of all proteins and their associated transcripts. The *x*-axis shows the mean value of the expressed protein in the group, and the *y*-axis indicates the mean value of the FPKM of the transcript in the same group. The expression of the transcript and the protein is taken as the logarithmic value. Each point represents a protein and its associated transcripts, rho in the legend represents the pearson correlation coefficient, *p* is the correlation test *p*-value. when rho > 0, it is called negative correlation; rho < 0, said positive correlation; rho = 0, called zero correlation, that is, no correlation; | rho| larger, the greater the correlation between the two groups. **(D)** The top 20 GO terms that contain the largest number of transcripts/proteins. Blue column indicates the transcripts in the transcriptome, orange column represents the proteins in the proteome. **(E)** The top 20 KEGG pathways that contains the largest number of transcripts/proteins. Blue column indicates the transcripts in the transcriptome, orange column represents the protein in the proteome. **(F)**. Venn diagram showing relevance relation between DEPs and DEGs.

As shown in **Figure 6A**, a total of 2352 genes were identified at both protein and transcript levels. However, only 72 DEPs were also detected in DEGs (**Figure 6B**). The Pearson correlation coefficient was -0.1542 (*p* < 0.01, **Figure 6C**), which indicated that the relative expression quantity of the genes at transcript level correlated with protein level positively but poorly. In addition, most of the genes were concentrated in the center of the coordinate, indicating that these genes were consistent at transcriptome and proteome levels. The GO analyses for both DEGs and DEPs demonstrated that the major GO terms were focused on “binding,” “metabolic process,” “catalytic activity,” “cellular process,” and “single-organism process,” respectively (**Figure 6D**). Furthermore, within the most significantly enriched pathways, most DEGs were amassed in “metabolic pathways,” “ribosome,” and “protein digestion and absorption,” while the major-enriched pathways in DEPs were “ribosome,” “ribosome biogenesis,” “RNA transport,” and “spliceosome” (**Figure 6E**).

Basing on the correlation analysis between transcriptomic and proteomic data, we detected 15 up-regulated and 16 down-regulated genes (with function annotation) in SF at both transcript and protein levels. These up-regulated genes in SF included *Amy2, Cryaa, Ddb_G0274169, Cdipt, hemoglobin, Vg, Vg2, Mettl10, Vat1L, Kcp, Hsp-16.2, Gpx3, N/A^a^, Rps26,* and *Sod1* (**Table 3**); while the down-regulated genes in SF included *Tsr, Sec5, Man2B1, N/A^d^, Smpd1, Etfa, Nup155, Ebna1Bp2, Lolal, Anpep, Zetatry, CpipJ_CPIJ014254, Rps17, Ugt2B31, Aael007945, and N/A^e^* (**Table 4**). To further dig out the related information on reproductive switching, the top 40 DEGs (Supplementary Table S6) and DEPs (Supplementary Table S7) were analyzed, respectively. Among these top DEPs, five proteins (Vg2, Mettl10, Vat1L, Cryas and Hsp-16.2) were significantly up-regulated at both protein and transcript levels (Supplementary Table S8), while there were no proteins to be down-regulated at both protein and transcript levels in this case. At the same time, we also found that some up-regulated proteins (e.g., Hemoglobin, sod, amy2 and rps26) showed a relatively low differential expression at the transcript level (Supplementary Table S9). In addition, some down-regulated proteins (e.g., clca4a, npc2, sgabd-1, sec5, keratin, krt8, ugt2b31, and man2b1) also displayed a relatively low differential expression at transcript level (Supplementary Table S10).

**Table 3:** Up-regulated genes in SF with the same trend at transcriptomic and proteomic levels.

Gene	T(FC^SP^/_PF_)	*P*-value	P(FC^SF^/_PF_)	*P*-value	Function
*Amy2*	23.95	4.46E-10	1.97	0.0024	Alpha-amylase activity
*Cryaa*	8091.64	8.87E-32	2.12	0.0087	Metal ion binding
*Ddb_G0274169*	56.58	3.05E-13	2.17	0.0019	Chitin binding
*Cdipt*	121.58	1.05E-16	1.65	0.0022	Cobalamin transport
*Hemoglobin*	3030.29	1.13E-24	2.89	0.0007	Iron ion binding/heme binding
*Vg*	449.32	6.10E-22	3.55	0.0020	Lipid transport
*Vg2*	26.47	1.95E-10	3.04	0.0025	Lipid transport
*Mettl10*	1759.13	2.19E-26	2.28	0.0007	Methyltransferase activity
*Vat1L*	4971.83	1.87E-30	2.15	0.0068	Oxidation-reduction process
*Kcp*	8.58	3.98E-06	1.61	0.0036	Protein binding
*Hsp-16.2*	1074.67	7.78E-22	2.11	0.0085	Response to heat
*Gpx3*	8.63	3.84E-06	1.73	0.0272	Response to oxidative stress
*N/A^a^*	507.88	7.06E-23	1.91	0.0113	Structural constituent of cuticle
*Rps26*	7.83	0.0000131	1.95	0.0018	Ribosome
*Sod1*	15.68	2.52E-08	3.78	0.4248	Superoxide metabolic process

*N/A^*a*^, Endocuticle structural glycoprotein SgAbd-8; T(FC^*PF*^/_*SF*_), the fold-changes in M. micrura (PF vs. SF) basing on the transcriptome analyses; P(FC^*PF*^/_*SF*_), the fold-changes in M. micrura (PF vs. SF) basing on the proteome analyses.*

**Table 4:** Up-regulated genes in PF with the same trend at transcriptomic and proteomic levels.

Gene	T(FC^PF^/_SF_)	*P*-value	P(FC^PF^/_SF_)	*P*-value	Function
*Tsr*	2531	1.44E-22	1.49	0.0005	Actin filament depolymerization
*Sec5*	12.84	2.06E-06	2.15	0.0058	Oocyte development
*Man2B1*	28.17	6.06E-09	2.00	0.0030	Hydrolase activity
*N/A^d^*	32.52	5.78E-09	1.86	0.0128	Hydrolase activity
*Smpd1*	9.55	2.41E-05	1.60	0.0007	Hydrolase activity
*Etfa*	4877	5.72E-24	1.66	0.0013	Lipid homeostasis
*Nup155*	41.45	3.54E-06	1.73	0.0485	Nucleocytoplasmic transport
*Ebna1Bp2*	599.7	1.65E-16	1.81	0.0084	Ribosome biogenesis
*Lolal*	177.5	1.73E-11	1.77	0.0006	Protein binding
*Anpep*	12.30	3.8E-06	1.85	0.0010	Metallopeptidase activity
*Zetatry*	22.26	1.19E-05	1.94	0.0037	Serine-type endopeptidase activity
*CpipJ_CPIJ014254*	18.16	6.43E-08	1.59	0.0003	Serine-type endopeptidase activity
*Rps17*	47.49	1.45E-06	1.53	0.0027	Structural constituent of ribosome
*Ugt2B31*	25.52	3.1E-08	2.12	0.0028	Transferase activity
*Aael007945*	511.5	7.67E-16	1.97	0.0050	Translation initiation factor activity
*N/A^e^*	12.11	2.09E-06	1.59	0.0023	Transporter activity

*N/A^*d*^, esterase FE4; N/A^*e*^, fatty acid-binding protein; T(FC^*PF*^/_*SF*_), the fold-changes in M. micrura (PF vs. SF) basing on the transcriptome analyses; P(FC^*PF*^/_*SF*_), the fold-changes in M. micrura (PF vs. SF) basing on the proteome analyses*.

The comparison between transcriptome and proteome displayed that the expression patterns of proteins showed a general correlation with their mRNA expression at transcript level. Furthermore, our data also revealed significantly discordant regulation between transcriptional and translational levels. We found 59 up-regulated (FC > 1.5) (Supplementary Table S11, with function annotation) and 469 down-regulated proteins in SF (Supplementary Table S12, FC > 1.8) at proteomic level but in absence of changes in mRNA expression at transcript level (**Figure 6F**). In addition, 4 up-regulated (Supplementary Table S13) and 11 down-regulated proteins (Supplementary Table S14) showed abundance changes in the opposite direction at transcript level (**Figure 6F**).

Besides transcriptional regulation, it is clear that gene expression is also modulated at post-transcriptional levels. To better highlighting the importance of post-transcriptional processes, we further identified 13 key reproductive-switching related DEPs in SF, including Vg fused with superoxide dismutase (Dmagvtg1), ferritin subunit (Ferh), cathepsin l-like proteinase (Cat-1), NADH dehydrogenase 1 alpha subcomplex assembly factor (Ndufaf5), chorion peroxidase (Pxt), cuticle protein 7 (N/A^c^), cytoglobin-2 (Cygb2), endocuticle structural glycoprotein SgAbd-2 (N/A^l^), probable cytochrome P450 301a1 (Cyp301a1), pupal cuticle protein 20 (Pcp20), heat shock protein ECU02_0100 (Ecu02_0100), ferritin heavy chain (N/A^m^), apolipoprotein D (Apod) (**Table 5**).

**Table 5:** The significantly up-regulated proteins and discordant regulation at mRNA level in *Moina micruras* (SF vs. PF).

Protein	FC(^SF^/_PF_)	*P*-value	Gene	FC(^SF^/_PF_)	FDR
Vitellogenin fused with superoxide dismutase	3.33	0.0001553	*Dmagvtg1*	1.04	1.0000000
Ferritin subunit	2.25	0.0060266	*Ferh*	0.13	0.0087660
Cathepsin L-like proteinase	2.01	0.0113644	*Cat-1*	1.91	0.3457043
NADH dehydrogenase 1 alpha subcomplex assembly factor	1.87	0.0076539	*Ndufaf5*	0.79	0.9444270
Chorion peroxidase	1.85	0.0023514	*Pxt*	0.59	0.7086529
Cuticle protein 7	1.83	0.0007276	*N/A^c^*	3.03	0.0821342
Cytoglobin-2	1.82	0.0101651	*Cygb2*	0.96	0.9646688
Endocuticle structural glycoprotein SgAbd-2	1.65	0.0239360	*N/A^l^*	0.00	1.0000000
Probable cytochrome P450 301a1	1.61	0.0073307	*Cyp301a1*	1.03	0.8814535
Pupal cuticle protein 20	1.60	0.0113674	*Pcp20*	0.64	0.7253701
Heat shock protein ECU02_0100	1.59	0.0025001	*Ecu02_0100*	0.35	0.4072912
Ferritin heavy chain	1.56	0.0068386	*N/A^m^*	0.12	0.0031195
Apolipoprotein D	2.13	0.0061593	*Apod*	0.09	0.0002418

*N/A^*c*^, cuticle protein 7; N/A^*l*^, endocuticle structural glycoprotein SgAbd-2; N/A^*m*^, ferritin heavy chain.*

### Quantitative PCR Analysis of Candidate Genes

To further confirm the transcriptomic and proteomic results, 21 DEGs or DEPs were randomly selected for validation by qRT-PCR. The results showed that the 11 up-regulated genes in SF (*Amy2, Cryaa, Ddb_Gg0274169, Hemoglobin, Vg, Vg2, Hsp-16.2, Gpx3, N/A^a^, Mettl10* and *Sod1*) were all confirmed by qRT-PCR (**Table 6**). In addition, the up-regulated genes in PF (*Tsr, Sec5, Man2B1, Nup155, Ebna1Bp2, Anpep, Zetatry, Rps17, Aael007945,* and *Ugt2B31*) were also confirmed by qRT-PCR (**Table 6**). The RNA-seq demonstrated that the expression level of *Nup155, Rps17* and *AaeI007945* genes in PF were higher than that in SF with the fold-changes: 41.15, 47.49, and 511.58, respectively, but the fold-changes detected by qRT-PCR were only 1.13, 1.35, and 1.33, respectively (**Table 6**).

**Table 6:** Verification Table for qPCR, RNA-seq and iTRAQ.

Fold Changes (SF Vs. PF)				Fold Changes (PF Vs. SF)			
Gene	qPCR	RNA-Seq	iTRAQ	Gene	qPCR	RNA-Seq	iTRAQ
*Amy2*	207.92	23.95	1.97	*Tsr*	2.49	2531.81	1.49
*Cryaa*	1271.46	8091.64	2.12	*Sec5*	3.75	12.84	2.15
*Ddb_G0274169*	15.56	56.58	2.17	*Man2B1*	8.38	28.17	2.00
*Hemoglobin*	286.26	3030.29	2.89	*Nup155*	1.13	41.15	1.73
*Vg*	274.71	449.32	3.55	*Ebna1Bp2*	1.61	599.72	1.81
*Vg2*	4.46	26.47	3.04	*Anpep*	2.03	12.30	1.85
*Hsp-16.2*	147.34	1074.67	2.11	*Zetatry*	349.9	22.26	1.94
*Gpx3*	8.22	8.63	1.73	*Rps17*	1.35	47.49	1.53
*SgAbd-8*	121.02	507.88	1.91	*Ugt2B31*	4.19	25.52	2.12
*Sod1*	51.94	15.68	3.78	*Aael007945*	1.33	511.58	1.97
*Mettl10*	70.95	1759.10	2.28				

*Verification table showing direction of change in gene expression with SF, compared with PF. The table represents selected genes of interest and compares similarities and differences in gene expression using qPCR, RNA-seq and Itraq*.

## Discussion

Similar to *Daphnia*, *M. micrura* could also switch their reproduction mode from parthenogenesis to sexual reproduction to adapt to the external environment. However, little is known about the molecular mechanisms of reproductive switching. In the present study, based on the transcriptomic and proteomic analyses, we tried to investigate DEGs and DEPs in *M. micrura* (SF vs. PF) and identify the critical genes involved in reproductive switching.

Gene ontology and KEGG analyses demonstrated that up-regulated and down-regulated genes in *M. micrura* (SF vs. PF) had different enrichment terms. The most frequently enriched terms in SF were cuticle (e.g., carbohydrate binding, polysaccharide binding, chitin binding, chitin metabolic process, and structural constituent of cuticle), hemoglobin (e.g., iron ion binding, heme binding and tetrapyrrole binding), and oxidative stress (e.g., oxidoreductase activity and oxidation-reduction process). Chitin is involved in cuticle formation in insects and is the most important barrier tissues against any environmental stresses (Pesch et al., 2015). Hemoglobin could allow the organisms to survive in low oxygen stress (Pirow et al., 2001). These results, taken together, suggest that the up-regulated genes in SF play an important role in producing a protective cuticle structure to resist unfavorable conditions, and increasing oxygen transport and storage in respond to hypoxia. In contrast, most basic metabolic processes (e.g., “metabolic process,” “primary metabolic process,” “macromolecule metabolic process,” and “protein metabolic process”) were the most frequently enriched groups in PF. As well known, PF can produce a large number of parthenogenetic embryos which could develop directly into PF without fertilization (Arbačiauskas, 2004), these results imply that up-regulation of various metabolic processes in SP might play an important role in nutrient uptake and further contribute to the growth of neonates.

Correlation analysis between transcriptomics and proteomics exhibit that the enrichment of proteins in different GO terms are consistent with the transcriptome results. However, the pathways enriched in proteins are especially grouped in genetic information processing, while various metabolic processes are dominated in transcriptome level. In addition, our present study demonstrates that 31 genes are significantly differentially expressed at both transcriptomic and proteomic levels, including 16 up-regulated genes and 15 down-regulated genes in SF. Although transcriptional and translational regulation are generally coordinated, we also identify a lot of genes that oppose this trend. A total of 543 DEPs in SF are showed differential expression pattern in transcript level, implying the involvement of post-transcriptional regulation. Previous study has also demonstrated that post-transcriptional processing determined steady-state protein levels (Chen and Harmon, 2006). To further explore the effects of post-transcriptional regulation in reproductive switching, we also dig out some proteins that went against the overall trend of concordant regulation at mRNA and protein levels, including Dmagvtg1, Ferh, Cat-1, Ndufaf5, Pxt, cuticle protein 7 (N/A^c^), Cygb2, endocuticle structural glycoprotein SgAbd-2 (N/A^l^), Cyp301a1, Pcp20, Ecu02_0100, ferritin heavy chain (N/A^m^), and Apod. These post-transcriptionally regulated genes may play a more important role in reproductive switching than those transcriptionally regulated genes in *M. micrura*. Finally, the critical genes and their protein products involved in reproductive switching are analyzed as follows.

### Oxygen-Transport Genes

The globins are a superfamily of heme-containing globular proteins, involved in binding and/or transporting oxygen, specifically for iron ion binding, heme binding, and oxygen transport (Baldi et al., 1994; Culbertson, 2010). In the present study, the globin members in SF were found to contain one significantly up-regulated gene (*hemoglobin*) at transcriptomic and proteomic levels and three significantly up-regulated genes (*Cygb2*, *Ferh* and *ferritin heavy chain*) at post-transcriptional level. The hemoglobin is one of the most important globin protein, which plays a key role in oxygen transport. In invertebrates, hemoglobin concentrations are low in most cases. However, hypoxia can cause a significantly increase in the hemoglobin concentrations of freshwater zooplankton organisms, which indicates that the induction of hemoglobin synthesis could improve the tolerance of the daphnids to low environmental dissolved oxygen levels, and allows the organisms to survive under this stress (Pirow et al., 2001). Given that environmental oxygen levels are relatively low in the occurrence of sexual reproduction in cladoceran, it is further demonstrated that the dissolved oxygen levels may be an important environmental factor for the reproduction switching of cladoceran. Similar to hemoglobin, the Cygb2 existing in proteomic data also belongs to the globin family, which is significantly up-regulated in SF. Since previous studies have demonstrated that Cygb2 could be up-regulated under hypoxic conditions and played a key role in oxygen-requiring reactions (Fago et al., 2004; Schmidt et al., 2004), we speculate that Cygb2 may be also involved in intracellular oxygen storage or transfer in *M. micrura*. In addition to *hemoglobin* and Cygb2, Ferh and ferritin heavy chain also played an important role in ferric iron binding and transport. Larade and Storey (2004) reported that the concentration of ferritin could be rapidly increased in response to stresses such as anoxia, which implies that ferritin is an acute phase protein for hypoxia (Beck et al., 2002). As we know, environmental signals could regulate a variety of key physiology processes in animals, the globin-related genes involved in oxygen transport were all up-regulated in SF in response to hypoxia. These results, taken together, indicate that low oxygen content may lead to reproductive switching of *M. micruras*. On the other hand, the high expression levels of the globin-related genes in SF may also indicate that SF needs more oxygen to finish sexual reproduction and to produce resting eggs.

### Vitellogenin-Related Genes

Vitellogenin (Vg/Vit-2) and vitellogenin-2 (Vg2) are both the members of the Vg family that play a key role in the formation of yolk proteins in cladocerans (Kato et al., 2004). Vg is the major precursor of the egg-yolk proteins, vitellins (Vn), which provide energy for embryonic development in oviparous organisms (Matozzo et al., 2008). In the present study, the expression levels of *Vg* gene and *vitellogenin-2* gene were both significantly higher in SF compared to PF. Similar results were also reported in *D. similoides* (Zhang et al., 2016). In addition, we also found that *Dmagvtg1* was significantly up-regulated in SF at post-transcriptional level. Previous study has reported that *Dmagvtg1* encoded Vg, and the precursor to Vg was the most abundant polypeptide in *D. magna* eggs (Kato et al., 2004). These results indicate that the SF could produce more vitellins to package into the resting eggs for embryonic development. In addition, similar to Vg, the *F40A3.3* was also highly expressed in SF, which was annotated as lipid transport or lipid transporter activity, and played an important role in lipid binding. Hence, it may be also involved in the synthesis of egg-yolk proteins in SF.

### Cuticle-Related Genes

In the present study, seven genes and their protein products (cuticle protein 6, cuticle protein 7, endocuticle structural glycoprotein SgAbd-2, endocuticle structural glycoprotein Sgabd-8, Pcp20, Pcp27, and Cyp301a1), related to the synthesis of cuticle or chitin and the formation of cuticle, were significantly up-regulated in SF compared to PF. Of these genes, the expression levels of cuticle protein 7, endocuticle structural glycoprotein SgAbd-2, Pcp20 and Cyp301a1 were found significantly higher in SF compared to PF at post-transcriptional level. The cuticle proteins and chitin are both involved in the formation of cuticle, which is the most important barrier against the environmental stresses in insects (Neville et al., 1976; Liu et al., 2014). In cladocerans, the cuticle also consists of cuticle protein and chitin, which can withstand adverse conditions of external environment (Repka et al., 1995; Liu et al., 2014). A recent study demonstrated that D. magna could develop an array of morphological changes in the presence of Triops cancriformis, including changes of carapace morphology and cuticle hardening (Otte et al., 2014). Besides, another study suggested that Cyp301a1 was involved in the formation of the adult cuticle in Drosophila melanogaster (Sztal et al., 2012). Taken together, the high expression of cuticle-related genes and proteins indicates that these genes could help SF to undergo a series of corresponding changes in cuticle structure as a response to the adverse external environmental conditions. Furthermore, the SF needs to produce resting eggs to survive in unfavorable environmental conditions, and those resting eggs are packed by a black ephippium with a thick cuticle. These findings indicate that the up-regulated cuticle-related genes in SF might also play a key role in the formation of ephippium.

### Hsp-Related Genes

In this study, three members of heat shock protein (Hsp)-related family (*Hsp-16.2, ECU02_0100,* and *Cryaa*) were also highly detected in SF. *Hsp-16.2* encodes a small heat shock protein (sHsp) which plays an important role in protecting proteins against stress-induced aggregation (Pozsgai et al., 2007). Previous study has demonstrated that the anti-apoptotic effect of *Hsp16.2* was mediated by the activation of Hsp90, with which *Hsp16.2* binds (Bellyei et al., 2007a). Furthermore, overexpression of Hsp16.2 can increase lipid raft formation, thus helping to stabilize the plasma membrane (Bellyei et al., 2007a,b). *ECU02_0100* is an Hsp70-related protein, which is a member of Hsp70s. Hsp70s play a vital role in stress tolerance and survival under adverse conditions (Mikulski et al., 2011). They can also promote the correct folding of newly synthesized proteins (Mayer and Bukau, 2005). *Cryaa* can encode alpha-crystallin A chain (αAC), which belongs to the alpha-crystallin (αC). Similar to Hsp16.2, αC has obvious sequence homology with the amino acid sequence of 90–100 residues at the C terminus of the sHSP family (Klemenz et al., 1991). It is also demonstrated that the expression of αC increase significantly under heat stress and oxidative stress, which suggests that the αC is also a molecular chaperone (similar to sHSP) in response to multiple environmental stresses. These findings, as a whole, indicate that the high expression of three *Hsp* genes in SF can protect the *M. micrura* against various environmental stress stimuli (e.g., low or high temperature, hypoxia) by maintaining cellular homeostasis. In addition, these findings also indicate that Hsp proteins may be the key proteins that deal with environmental stresses and maintain the homeostasis, which play an important role in the success of reproductive switching.

### Methyltransferase-Related Genes

Methyltransferases are a large group of enzymes including protein methyltransferases, DNA/RNA methyltransferase, natural product methyltransferases, and non-*S*-Adenosyl methionine (SAM) dependent methyltransferase. For the protein methyltransferase, previous studies have demonstrated that farnesoic acid *O*-methyltransferase was a major contributing enzyme in the synthesis of methyl farnesoate in decapod and branchiopod crustaceans (Ruddell et al., 2003; Li et al., 2010). Recent study has reported that methyl farnesoate played a key role in regulating the reproductive switching in water flea (LeBlanc and Medlock, 2015). These findings suggest that the up-regulated genes encoding methyltransferases (*Mettl10* and *Ndufaf5*) in SF may also be related to the reproductive switching in *M. micrura*. On the other hand, the DNA methyltransferase can catalyze the transfer of a methyl group to DNA. DNA methylation, a key component of genetic regulation, occurs primarily at the 5-carbon of the base cytosine, forming 5′ methylcytosine, and does not entail a change in DNA sequences (Lan et al., 2010). The presence of DNA methylation has been detected in *D. magna*, which suggested that potentially epigenetic effect might occur in this species (Vandegehuchte et al., 2009). In this study, the *Mettl10* and *Ndufaf5* gene products were both highly detected in SF, which suggested that the DNA methylation might also be related to the reproductive switching in *M. micrura*.

### Genes Encoding Some Peptidases, Peroxidase and Lipocalin

Our iTRAQ results showed that the genes encoding some peptidases, peroxidase and lipocalin were all highly detected in SF compared to PF. Cathepsin L-like proteinase was up-regulated in *Helicoverpa armigera* during larval molting (Wang et al., 2010). Chorion peroxidase was involved in the formation of a rigid and insoluble egg chorion in *Aedes aegypti* (Li et al., 1996). We speculate that both cathepsin L-like proteinase and chorion peroxidase may be involved in the development of embryos and juveniles under adverse condition in *M. micrura*. Apolipoprotein D was an acute response protein with a protective and was involved in the mechanisms regulating protection from oxidative stress (Ganfornina et al., 2008). In the present study, apolipoprotein D was also significantly up-regulated in SF, which may contribute to protecting *M. micrura* from oxidative stress. These findings, taken together, suggest that the up-regulated cathepsin L-like proteinase, chorion peroxidase and apolipoprotein D in SF may play a role in reproductive switching in *M. micrura*.

In summary, we identified several critical DEGs and DEPs involved in reproductive switching in *M. micruras* basing on the analysis of transcriptome and proteome. We found post-transcriptional regulation may play an important role in reproductive switching in *M. micrura.* Those top up-regulated genes and their protein products in SF mainly belonged to globin-related family, Vg-related family, cuticle-related family, Hsp-related family and methyltransferase-related family, which indicates that these genes may play a key role in the reproductive switching in *M. micrura*. In contrast, the clusters of orthologous groups reveal that up-regulated genes in PF are strongly associated with the metabolic process, which may be responsible for rapid population proliferation of PF. Our comprehensive transcriptome and proteome data provide a valuable resource and offer novel insights into understanding post-transcriptional regulation in reproductive switching in *M. micrura*. Furthermore, this study also provides many key candidate genes and their protein products related to reproductive switching for further functional studies in *M. micrura*.

## Author Contributions

XL and GH conceived the project. JJ, LL, and CL performed the experiments. YD and GW contributed to the sample collection. JJ and GH conducted the bioinformatics analysis. JJ, XL, and GH contributed to the manuscript preparation.

## Conflict of Interest Statement

The authors declare that the research was conducted in the absence of any commercial or financial relationships that could be construed as a potential conflict of interest.

## Footnotes

1 http://www.ncbi.nlm.nih.gov/Traces/sra

2 https://github.com/jstjohn/SeqPrep

3 https://github.com/najoshi/sickle

4 http://trinityrnaseq.sourceforge.net/

5 http://www.iprox.org/

## Abbreviations

DEGs: differentially expressed genes
DEPs: differentially expressed proteins
iTRAQ: isobaric tag for relative absolute quantitation
PF: parthenogenetic female
SF: sexual female
Vg: vitellogenin.
