# Supplementary material for: Transcriptional and Translational Relationship in Environmental Stress: RNAseq and ITRAQ Proteomic Analysis Between Sexually Reproducing and Parthenogenetic Females in Moina micrura
Source: Front Physiol. 2018 Jul 2;9:812. doi: 10.3389/fphys.2018.00812 (PMC6036137; doi:10.3389/fphys.2018.00812)
Supplement: Supplementary file 1 [file Table_1.DOCX]

**Supplemental Table S1. Primers used in the RT-PCR assays**

| **Gene** | **Forward (5'-3')** | **Reverse (5'-3')** | **Annealing Tm** | **Production**  **Size (bp)** |
| --- | --- | --- | --- | --- |
| *Tsr* | CCACCAATGTCAAGGAACTCAAG | CTTCTCTTCAACAGCCTCAGGA | 65℃ | 199 |
| *Sec5* | CGCCAAATACGGAAAGCCTACTC | GATTCTTGTGTTCGGTTGCTGTTG | 66℃ | 212 |
| *Man2B1* | GCCGCACATTTCTTTCATTCCAA | GACAACGCCGCATCTTCTCTT | 64℃ | 192 |
| *Nup155* | TCCAAGTGCTGCGAGAAGACC | CGGAAACGGGAGAACTGAACG | 62℃ | 237 |
| *Ebna1Bp2* | GAATGGGTGGAACGCCTTGAC | CCTTCAGCCTTGCCAATCCTTC | 65℃ | 196 |
| *Anpep* | GAACTGGACGACGAATGGATACG | AGAGGGCGGGAGACTTGAATC | 64℃ | 207 |
| *Zetatry* | TCGTGCGGTGGGTCTATTCTG | ATGTCGTTCTCGTCGGTCCTC | 64℃ | 200 |
| *Rps17* | CATTATCCCAAGCAAACCTCTG | GGGTCAATCTCAATCGTGTCAA | 62℃ | 183 |
| *Ugt2B31* | AACGCACGGAGGATTGAACAG | TTGGCAGCATAAATCGGGTTGT | 65℃ | 207 |
| *Aael007945* | ACCTTGTGAATGCCTTGCTTTG | TGCTTCTCTTGTTGCTGCTTGA | 66℃ | 199 |
| *Amy2* | TCAGACAGCAACTACCGCACAC | CATTCGGAGCATTGGCAGCATT | 64℃ | 195 |
| *Cryaa* | ATTTGGCGGATTCGGTGGACTG | CTCGGGACGGAACTGGGATACA | 62℃ | 223 |
| *Ddb_G0274169* | GACGATTCGCTTACTTGATTG | TTCAAACGAGGGCACATAG | 64℃ | 205 |
| Hemoglobin | CACTGACTTGAACACTGGTATG | TCCGTATCCGCTCTGGTAAC | 66℃ | 223 |
| *Vg* | CCTACAAGCAGGCTATGGA | CGTGGAACTGCTTACCCTT | 65℃ | 190 |
| *Vg2* | AGGAGGAGGAAGAAGAAGAGGAA | GGCGGATTGCTGACGAACA | 63℃ | 223 |
| *Hsp-16.2* | CGTGCGACAAGCCGTAAAA | CAAGAGTTGCGTGGGGTAAAG | 64℃ | 212 |
| *Gpx3* | ATTCCGCAAGATAGAGGTCAATGG | CTGGCGTCGTAGCGTTTCACT | 63℃ | 201 |
| *N/A^a^* | CAAGCAGAAGTTTACAAGCAC | GCCCGACTCGTATCCGTAT | 66℃ | 211 |
| *Sod1* | GCACAACTCTTCCCGTATTCAACT | AGCCTCAGTCTCATAGCCGAAC | 65℃ | 206 |
| *Mettl10* | AACTGATATGGAGAAGCAATACGAG | CATCGGTGTAGCCTAAGTGGAA | 64℃ | 206 |
| *GAPDH* | GTCGCTTGGTGCTCCGTG | CGTTCGCTGAAGACCTGGAT | 64℃ | 193 |
