# Supplementary material for: Transcriptional and Translational Relationship in Environmental Stress: RNAseq and ITRAQ Proteomic Analysis Between Sexually Reproducing and Parthenogenetic Females in Moina micrura
Source: Front Physiol. 2018 Jul 2;9:812. doi: 10.3389/fphys.2018.00812 (PMC6036137; doi:10.3389/fphys.2018.00812)
Supplement: Supplementary file 2 [file Table_2.DOCX]

**Supplemental Table S2**

**The up-regulated genes in *Moina micruras* (SF vs. PF).**

| **Gene** | **FC^SF^/_PF_** | **FDR** | **Function** | **Gene** | **FC^SF^/_PF_** | **FDR** | **Function** |
| --- | --- | --- | --- | --- | --- | --- | --- |
| *Sls* | 244.1 | 5.73E-11 | Actin binding | *Sdha* | 950.9 | 3.50E-16 | Oxidoreductase activity |
| *Xpnpep3* | 134.3 | 9.45E-09 | Aminopeptidase activity | *Ermp1* | 115.2 | 3.57E-08 | Peptidase activity |
| *Abcb1A* | 168.5 | 1.41E-09 | ATP binding | *Ncln* | 194.9 | 4.02E-10 | Peptidase activity |
| *Bckdk* | 273.9 | 9.23E-14 | ATP binding | *Ppif* | 119.5 | 2.80E-09 | Peptidyl-prolyl cis-trans isomerase activity |
| *Atp5d* | 284.3 | 1.49E-11 | ATPase activity | *Dut* | 172.3 | 1.17E-09 | Peroxisome proliferator activated receptor |
| *Svep1* | 301.5 | 8.76E-12 | Calcium ion binding | *Gk* | 265.6 | 2.66E-11 | Phosphotransferase activity |
| *Lgals6* | 15666.7 | 1.64E-13 | Carbohydrate binding | *Aamp* | 411.8 | 5.38E-13 | Protein binding |
| *Ca14* | 188.9 | 3.47E-14 | Carbonate dehydratase activity | *Alx-1* | 737.4 | 8.60E-18 | Protein binding |
| *Ca2* | 114.0 | 3.10E-07 | Carbonate dehydratase activity | *Cntnap4* | 255.0 | 3.37E-10 | Protein binding |
| *Cahz* | 141.1 | 1.39E-15 | Carbonate dehydratase activity | *Cryaa* | 8091.6 | 5.40E-27 | Protein binding |
| *Acsbg2* | 809.8 | 1.75E-16 | Catalytic activity | *Dcaf11* | 435.7 | 3.17E-13 | Protein binding |
| *Tpsb* | 1235.4 | 6.40E-12 | Catalytic activity | *Fkbp4* | 103.5 | 7.72E-08 | Protein binding |
| *Spg21* | 311.7 | 6.40E-12 | CD4 receptor binding | *Fsn* | 205.0 | 2.70E-10 | Protein binding |
| *Chit1* | 312.3 | 1.94E-14 | Chitin binding | *Mel-26* | 530.0 | 5.86E-14 | Protein binding |
| *Cht3* | 602.4 | 8.80E-13 | Chitin binding | *Nemp1* | 244.4 | 5.73E-11 | Protein binding |
| *Cdc5L* | 390.2 | 4.12E-14 | Chromatin binding | *Ryk* | 239.9 | 6.48E-11 | Protein binding |
| *Cdipt* | 121.6 | 4.12E-14 | Cobalamin binding | *Sfrp5* | 339.8 | 3.02E-12 | Protein binding |
| *Cers6* | 139.5 | 6.65E-09 | DNA binding | *Sgf29* | 138.4 | 7.44E-09 | Protein binding |
| *Gcm* | 272.2 | 2.21E-11 | DNA binding | *Spopl* | 206.0 | 3.18E-10 | Protein binding |
| *H2A.F/Z* | 2058.8 | 3.04E-19 | DNA binding | *Stxbp5* | 5500.0 | 1.63E-09 | Protein binding |
| *H4* | 7878.8 | 1.53E-13 | DNA binding | *Tdpoz5* | 2640.0 | 3.31E-20 | Protein binding |
| *Lrrn2* | 470.4 | 1.64E-13 | DNA binding | *Ubxn4* | 655.0 | 9.10E-15 | Protein binding |
| *Mcm4* | 884.4 | 6.36E-16 | DNA binding | *Wdr82* | 106.1 | 6.66E-08 | Protein binding |
| *Mnd1* | 199.6 | 3.29E-10 | DNA binding | *Tom40* | 109.3 | 5.35E-08 | Protein channel activity |
| *Osa* | 210.0 | 2.12E-10 | DNA binding | *Bmpr2* | 799.3 | 1.55E-15 | Protein kinase activity |
| *Rtf1* | 898.3 | 5.50E-16 | DNA binding | *Ephb2* | 214.3 | 1.83E-10 | Protein kinase activity |
| *Psma3L* | 164.9 | 1.63E-09 | Endopeptidase activity | *Ksr2* | 246.9 | 5.23E-11 | Protein kinase activity |
| *Ddx54* | 214.0 | 1.83E-10 | Estrogen receptor binding | *Scyl1* | 327.3 | 4.29E-12 | Protein kinase activity |
| *Mftc* | 123.4 | 1.97E-08 | Folic acid transporter activity | *Tpk3* | 228.1 | 1.03E-10 | Protein kinase activity |
| *Fuctc* | 212.2 | 1.97E-10 | Fucosyltransferase activity | *Pebp1* | 195.4 | 4.02E-10 | Protein kinase binding |
| *Gdpgp1* | 402.7 | 6.48E-13 | GDP-D-glucose phosphorylase activity | *Anapc4* | 176.7 | 8.86E-10 | Protein phosphatase binding |
| *Mgst3* | 925.1 | 4.32E-16 | Glutathione transferase activity | *Ipo7* | 236.2 | 7.43E-11 | Ran GTPase binding |
| *Hcrtr1* | 102.0 | 9.01E-08 | G-protein coupled receptor activity | *Mew* | 555.3 | 3.96E-14 | Receptor activity |
| *Tkr86C* | 112.1 | 4.67E-08 | G-protein coupled receptor activity | *Hsp-16.2* | 1074.7 | 9.99E-09 | Response to heat |
| *Nudt20* | 252.0 | 4.33E-11 | Hydrolase activity | *Edc3* | 3319.0 | 9.99E-09 | RNA binding |
| *Nudt9* | 140.7 | 6.30E-09 | Hydrolase activity | *Fxr2* | 126.5 | 9.20E-14 | RNA binding |
| ***Pacs2*** | 170.5 | 1.23E-09 | Hydrolase activity | *Slbp* | 352.2 | 2.23E-12 | RNA binding |
| Hemoglobin | 3030.3 | 2.74E-21 | Iron ion binding | *Prss1* | 903.9 | 5.22E-16 | Serine-type endopeptidase activity |
| *F40A3.3* | 133.0 | 9.99E-09 | Lipid binding | *Serpinb1* | 825.0 | 1.18E-15 | Serine-type endopeptidase inhibitor |
| *Vg* | 449.3 | 7.27E-19 | Lipid transport | *Sxl* | 246.2 | 5.40E-11 | Sex determination |
| *Mapk14* | 246.8 | 5.23E-11 | MAP kinase activity | *Slc9A2* | 154.6 | 2.82E-09 | Solute:proton antiporter activity |
| *Atp5S* | 660.9 | 8.50E-15 | Metal ion binding | *N/A^a^* | 507.9 | 1.02E-19 | Structural constituent of cuticle |
| *Bco1* | 434.6 | 3.22E-13 | Metal ion binding | *N/A^b^* | 155.9 | 2.69E-09 | structural constituent of cuticle |
| *Plekhf2* | 350.0 | 2.24E-13 | Metal ion binding | *N/A^c^* | 2034.2 | 1.66E-23 | structural constituent of cuticle |
| *Mettl10* | 1759.1 | 7.40E-23 | Methyltransferase activity | *Pcp27* | 142.3 | 2.23E-09 | Structural constituent of cuticle |
| *Map4* | 130.0 | 1.26E-08 | Microtubule binding/tubulin binding | *Sod1* | 182.4 | 7.20E-10 | Superoxide dismutase activity |
| *Lamp5* | 241.6 | 6.12E-11 | Molecular function | *Atoh8* | 100.4 | 1.14E-07 | Transcription factor activity |
| *Myg1* | 131.1 | 1.12E-08 | Molecular function | *Crebzf* | 247.6 | 7.53E-16 | Transcription factor activity |
| *Rft1* | 155.0 | 2.82E-09 | Molecular function | *Nfyc* | 411.1 | 5.47E-13 | Transcription factor activity |
| *Rmnd1* | 375.6 | 1.21E-12 | Molecular function | *Taf5L* | 1910.3 | 6.15E-19 | Transcription factor activity |
| *Tchp* | 145.0 | 5.06E-09 | Molecular function | *Znfx1* | 1304.6 | 1.99E-17 | Transcription factor activity |
| *Prm_3* | 288.4 | 1.32E-11 | Motor activity | *Lpgat1* | 328.6 | 4.10E-12 | Transferase activity |
| *Rdh11* | 231.1 | 9.20E-11 | NADP-retinol dehydrogenase activity | *Pigm* | 598.0 | 2.04E-14 | Transferase activity |
| *Npfr* | 400.2 | 6.90E-13 | Neuropeptide F receptor activity | *Sord* | 492.7 | 1.07E-13 | Transferase activity |
| *Hrb87F* | 101.3 | 1.05E-07 | Nucleic acid binding | *Gtf2H2* | 180.9 | 7.51E-10 | Zinc ion binding |
| *Rsf1* | 319.5 | 5.35E-12 | Nucleic acid binding | *Ref(2)P* | 2515.0 | 5.24E-20 | Zinc ion binding |
| *Tmem63A* | 905.7 | 5.17E-16 | Nucleotide binding | *Rnf145* | 290.4 | 1.25E-11 | Zinc ion binding |
| *Uqcrb* | 670.5 | 7.36E-15 | Oxidation-reduction process | *Sord* | 143.7 | 5.34E-09 | Zinc ion binding |
| *Akr1A1* | 142.1 | 2.66E-11 | Oxidoreductase activity | *Vat1L* | 4971.8 | 3.78E-26 | Zinc ion binding |
| *Hsd17B12* | 1879.3 | 6.24E-21 | Oxidoreductase activity | *Zdhhc14* | 238.1 | 6.96E-11 | Zinc ion binding |

Note: *N/A^a^*: Endocuticle structural glycoprotein SgAbd-8; *N/A^b^*: Cuticle protein 6; *N/A^c^*: Cuticle protein 7
