# Supplementary material for: Transcriptional and Translational Relationship in Environmental Stress: RNAseq and ITRAQ Proteomic Analysis Between Sexually Reproducing and Parthenogenetic Females in Moina micrura
Source: Front Physiol. 2018 Jul 2;9:812. doi: 10.3389/fphys.2018.00812 (PMC6036137; doi:10.3389/fphys.2018.00812)
Supplement: Supplementary file 3 [file Table_3.DOCX]

**Supplemental Table S3**

**Most differentially down-regulated genes in SF (higher expression level in PF).**

| **Gene** | **FC^PF^/_SF_** | **FDR** | **Function** | **Gene** | **FC^PF^/_SF_** | **FDR** | **Function** |
| --- | --- | --- | --- | --- | --- | --- | --- |
| *Pfk* | 629.5 | 8.58E-11 | 6-phosphofructokinase activity | *Atxn3* | 226.0 | 3.41E-10 | Omega peptidase activity |
| *Pfe0165W* | 2531.8 | 1.95E-19 | Actin binding | *Rdh13* | 206.9 | 5.95E-09 | Oxidoreductase activity |
| *Ena* | 270.4 | 7.20E-11 | Actin binding | *Gk* | 359.1 | 5.81E-12 | Phosphotransferase activity |
| *Cat2* | 45.7 | 1.17E-08 | Amino acid transporter activity | *Ebp2* | 599.7 | 6.13E-14 | Poly(A) RNA binding |
| *Slc7A6* | 122.1 | 5.75E-08 | Amino acid transporter activity | *Rpusd3* | 118.0 | 7.72E-08 | Poly(A) RNA binding |
| *Slc25A16* | 95.3 | 5.87E-10 | Antiporter activity | *Tra2B* | 54.6 | 9.34E-09 | Poly(A) RNA binding |
| *Khc* | 150.6 | 9.99E-09 | ATP binding | *Wbp11* | 119.4 | 7.17E-08 | Poly(A) RNA binding |
| *Clca2* | 149.9 | 1.06E-08 | Calcium activated chloride channel | *Galt* | 220.5 | 4.37E-10 | Protein binding |
| *Clca4* | 40.1 | 3.14E-08 | Calcium activated chloride channel | *Aspm* | 296.9 | 3.12E-11 | Protein binding |
| *Mlc1* | 2946.5 | 1.07E-22 | Calcium ion binding | *Bap60* | 7824.8 | 4.22E-14 | Protein binding |
| *Svep1* | 335.4 | 1.09E-11 | Calcium ion binding | *Etfa* | 4877.2 | 1.05E-20 | Protein binding |
| *Lgals6* | 507.0 | 2.59E-13 | Carbohydrate binding | *Fbxo45* | 210.0 | 6.33E-10 | Protein binding |
| *Svep1* | 225.4 | 3.55E-10 | Carbohydrate binding | *Lolal* | 177.5 | 2.56E-09 | Protein binding |
| *Cat* | 275.0 | 1.46E-12 | Catalase activity | *Nemp1* | 199.9 | 9.24E-10 | Protein binding |
| *Odc1* | 1960.7 | 1.56E-18 | Catalytic activity | *Nfkbil1* | 116.5 | 9.01E-08 | Protein binding |
| *Seld* | 165.8 | 4.54E-09 | Catalytic activity | *Robo1* | 123.1 | 5.35E-08 | Protein binding |
| *Spg21* | 285.3 | 4.60E-11 | CD4 receptor binding | *Strn3* | 144.2 | 1.53E-08 | Protein binding |
| *Best2* | 114.8 | 9.73E-08 | Chloride channel activity | *Stxbp5* | 230.0 | 2.92E-10 | Protein binding |
| *Ctsh* | 54.9 | 1.31E-07 | Cysteine-type peptidase activity | *Tbl1Xr1* | 133.3 | 2.92E-08 | Protein binding |
| *Ctsl* | 257.5 | 9.20E-14 | Cysteine-type peptidase activity | *Atoh8* | 644.8 | 2.21E-09 | Protein dimerization activity |
| *Osa* | 180.0 | 2.32E-09 | DNA binding | *Glipr2* | 79.8 | 4.70E-09 | Protein homodimerization activity |
| *Rfx7* | 205.6 | 7.51E-10 | DNA binding | *Mmd2* | 1422.5 | 2.85E-17 | Protein kinase activity |
| *Ewg* | 188.3 | 1.63E-09 | DNA binding | *Bcl2L1* | 217.4 | 4.71E-10 | Protein kinase binding |
| *Trypsin-1* | 215.9 | 4.92E-10 | Erine-type endopeptidase activity | *Map2K1* | 189.1 | 5.09E-12 | Protein serine kinase activity |
| *Cg5065* | 126.7 | 1.06E-08 | Fatty-acyl-CoA reductase activity | *Dusp10* | 328.9 | 1.25E-11 | Protein serine phosphatase activity |
| *Gpx5* | 1079.0 | 3.60E-16 | Glutathione peroxidase activity | *Mew* | 220.2 | 4.07E-10 | Receptor activity |
| *Uvop* | 169.1 | 3.89E-09 | G-protein coupled receptor activity | *Cg13690* | 161.0 | 5.95E-09 | RNA binding |
| *Garnl3* | 205.4 | 7.82E-10 | GTPase activator activity | *Fxr1* | 165594.5 | 1.38E-20 | RNA binding |
| *Gna13* | 282.1 | 4.90E-11 | GTPase activity | *Nop60B* | 123.7 | 5.35E-08 | RNA binding |
| *Rab40C* | 342.5 | 8.96E-12 | GTPase activity | *Sars* | 307.2 | 2.35E-11 | Serine-tRNA ligase activity |
| *Tuba1C* | 201.8 | 8.46E-10 | GTPase activity | *Cpij014254* | 83.4 | 2.05E-10 | Serine-type endopeptidase activity |
| *Tufm* | 185.2 | 1.80E-09 | GTPase activity | *Cpij019031* | 82.9 | 1.84E-11 | Serine-type endopeptidase activity |
| *Mpg* | 101.0 | 2.76E-07 | Hydrolase activity | *Mbtps1* | 102.1 | 2.54E-07 | Serine-type endopeptidase activity |
| *Xpnpep1* | 158.9 | 6.65E-09 | Hydrolase activity | *Resilin* | 38732.5 | 3.02E-22 | Structural constituent of cuticle |
| *Shab* | 198.0 | 5.28E-10 | Ion channel activity | *Clc* | 287.3 | 4.33E-11 | Structural molecule activity |
| *Exl-1* | 106.5 | 1.69E-07 | Ion channel activity | *Ceh-9* | 142.9 | 1.62E-08 | Transcription factor activity |
| *Scarb1* | 37.5 | 4.96E-08 | Lipopolysaccharide binding | *Gabpa* | 371.9 | 4.29E-12 | Transcription factor activity |
| *Pomt2* | 109.2 | 1.57E-07 | Mannosyltransferase activity | *Nus1* | 101.5 | 2.54E-07 | Transferase activity |
| *Plekhf2* | 580.0 | 8.17E-14 | Metal ion binding | *Pigm* | 698.0 | 1.63E-14 | Transferase activity |
| *Pcgf3* | 439.4 | 9.61E-13 | Metal ion binding | *Eif3H* | 511.6 | 2.38E-13 | Translation initiation factor activity |
| *Dync1Li1* | 333.7 | 1.12E-11 | Microtubule motor activity | *Slc26A11* | 184.7 | 1.80E-09 | Transmembrane transporter activity |
| *Cg8135* | 369.2 | 4.64E-12 | Molecular function | *Tret1_26* | 61.6 | 3.09E-09 | Transmembrane transporter activity |
| *Cox20* | 147.0 | 1.26E-08 | Molecular function | *Sec14L2* | 104.9 | 4.45E-09 | Transporter activity |
| *Mfsd11* | 1617.8 | 9.18E-18 | Molecular function | *Slc15A2* | 136.3 | 2.40E-08 | Transporter activity |
| *Rmnd1* | 47.0 | 4.36E-08 | Molecular function | *Nsun2* | 1450.7 | 2.42E-17 | tRNA-methyltransferase activity |
| *Saysd1* | 163.0 | 5.34E-09 | Molecular function | *Spopla* | 47.6 | 1.28E-09 | Ubiquitin protein ligase binding |
| *Ndufb2* | 119.2 | 7.17E-08 | NADH dehydrogenase activity | *Slc39A1* | 168.1 | 4.09E-09 | Zinc ion transporter activity |
| *Exd1* | 118.3 | 7.72E-08 | Nucleic acid binding | *Anpep* | 120.8 | 6.17E-08 | Zinc ion binding |
| *Gpkow* | 308.6 | 2.21E-11 | Nucleic acid binding | *Cdd* | 205.5 | 7.82E-10 | Zinc ion binding |
| *Tardbp* | 130.8 | 3.34E-08 | Nucleic acid binding | *Pias1* | 133.2 | 2.92E-08 | Zinc ion binding |
| *Atpalpha* | 480.7 | 4.23E-13 | Nucleotide binding | *Zdhhc8* | 138.0 | 2.10E-08 | Zinc ion binding |
| *Pla2G15* | 164.1 | 5.06E-09 | O-acyltransferase activity |  |  |  |  |
