# Supplementary material for: Transcriptional and Translational Relationship in Environmental Stress: RNAseq and ITRAQ Proteomic Analysis Between Sexually Reproducing and Parthenogenetic Females in Moina micrura
Source: Front Physiol. 2018 Jul 2;9:812. doi: 10.3389/fphys.2018.00812 (PMC6036137; doi:10.3389/fphys.2018.00812)
Supplement: Supplementary file 4 [file Table_4.DOCX]

**Supplemental Table S4**

**Most differentially up-regulated proteins in SF compared to PF.**

| **Gene** | **FC^SF^/_PF_** | **P-value** | **Function** | **Gene** | **FC^SF^/_PF_** | **P-value** | **Function** |
| --- | --- | --- | --- | --- | --- | --- | --- |
| *Minpp1* | 1.52 | 0.0482 | Acid phosphatase activity | *Cyp301A1* | 1.61 | 0.0073 | Oxidation-reduction process |
| *Mp20* | 1.97 | 0.0002 | Actomyosin structure organization | *Sod1* | 3.78 | 0.0029 | Oxidation-reduction process |
| *Capsl* | 1.59 | 0.0025 | Calcium ion binding | *Vat1L* | 2.15 | 0.0068 | Oxidation-reduction process |
| *Mlc1* | 1.67 | 0.0204 | Calcium ion binding | *Decr1* | 1.54 | 0.0223 | Oxidoreductase activity |
| *Amy2* | 1.97 | 0.0024 | Carbohydrate catabolic process | *Sdr16C6* | 1.52 | 0.0044 | Oxidoreductase activity |
| *Bcat1* | 1.71 | 0.0024 | Catalytic activity | *Apod* | 2.13 | 0.0062 | Pigment binding |
| *G0274169* | 2.17 | 0.0019 | Chitin binding | *Ga18377* | 2.26 | 0.0076 | Protein binding |
| *Cdipt* | 1.65 | 0.0022 | Cobalamin binding | *Kcp* | 1.61 | 0.0036 | Protein binding |
| *Cat-1* | 2.01 | 0.0114 | Cysteine-type peptidase activity | *Notch3* | 1.62 | 0.0088 | Protein binding |
| *Ndufb3* | 1.79 | 0.0069 | Electron transport chain | *Sls* | 2.59 | 0.0009 | Protein binding |
| *Ferh* | 2.25 | 0.0060 | Ferric iron binding | *Smtnl1* | 2.57 | 0.0021 | Protein binding |
| *Gvin1* | 1.77 | 0.0463 | GTPase activity | *Dusp3* | 1.60 | 0.0086 | Protein dephosphorylation |
| *Tef1* | 1.62 | 0.0026 | GTPase activity | *Ap2S1* | 1.92 | 0.0014 | Protein transporter activity |
| *Cg2145* | 1.87 | 0.0004 | Hydrolase activity | *Hsp-16.2* | 2.11 | 0.0085 | Response to heat |
| Hemoglobin | 2.89 | 0.0007 | Iron ion binding/heme binding | *Ecu02_0100* | 1.59 | 0.0025 | Response to heat |
| *Cygb2* | 1.82 | 0.0102 | Iron ion binding/heme binding | *Gpx3* | 1.73 | 0.0272 | Response to oxidative stress |
| *Vg* | 3.55 | 0.0020 | Lipid transport | *Pxt* | 1.85 | 0.0024 | Response to oxidative stress |
| *Vg2* | 3.04 | 0.0025 | Lipid transport | *Slc6A1* | 1.73 | 0.0186 | Sodium symporter activity |
| *Cpa2* | 1.80 | 0.0041 | Metallocarboxypeptidase activity | *Pcp20* | 1.60 | 0.0114 | Structural constituent of cuticle |
| *Mmel1* | 1.68 | 0.0002 | Metalloendopeptidase activity | *Rpl15* | 1.64 | 0.0213 | Structural constituent of ribosome |
| *Mettl10* | 2.28 | 0.0007 | Methyltransferase activity | *Rpl23P* | 1.74 | 0.0058 | Structural constituent of ribosome |
| *Ndufaf5* | 1.87 | 0.0077 | Methyltransferase activity | *Rpl27C* | 2.55 | 0.0063 | Structural constituent of ribosome |
| *Spn-E* | 1.68 | 0.0016 | Nucleic acid binding | *Rps14A* | 1.78 | 0.0020 | Structural constituent of ribosome |
| *Apt1* | 1.53 | 0.0020 | Nucleoside metabolic process | *Rps26* | 1.95 | 0.0018 | Structural constituent of ribosome |
| *odf* | 3.33 | 0.0002 | Oxidation-reduction process | *Htb1* | 2.00 | 0.0083 | Transferase activity |
