# Supplementary material for: Transcriptional and Translational Relationship in Environmental Stress: RNAseq and ITRAQ Proteomic Analysis Between Sexually Reproducing and Parthenogenetic Females in Moina micrura
Source: Front Physiol. 2018 Jul 2;9:812. doi: 10.3389/fphys.2018.00812 (PMC6036137; doi:10.3389/fphys.2018.00812)
Supplement: Supplementary file 5 [file Table_5.DOCX]

**Supplemental Table S5**

**Most differentially down-regulated proteins in SF compared to PF.**

| **Gene** | **FC^PF^/_SF_** | **P-value** | **Function** | **Gene** | **FC^PF^/_SF_** | **P-value** | **Function** |
| --- | --- | --- | --- | --- | --- | --- | --- |
| *Sms* | 1.63 | 0.0025 | Amino acid metabolic process | *G3Bp2* | 1.67 | 0.0057 | Nucleic acid binding |
| *Cadn* | 1.82 | 0.0124 | Calcium ion binding | *Htatsf1* | 1.72 | 0.0139 | Nucleic acid binding |
| *Calua* | 1.94 | 0.0033 | Calcium ion binding | *Me31B* | 1.92 | 0.0006 | Nucleic acid binding |
| *Lrp1* | 1.65 | 0.0005 | Calcium ion binding | *Nkrf* | 1.68 | 0.0053 | Nucleic acid binding |
| *Lrp2* | 1.61 | 0.0041 | Calcium ion binding | *Rbm5* | 1.70 | 0.0076 | Nucleic acid binding |
| *Amy2* | 1.61 | 0.0305 | Carbohydrate metabolic process | *Supt6H* | 1.95 | 0.0172 | Nucleic acid binding |
| *Amy58* | 1.64 | 0.0024 | Carbohydrate metabolic process | *Ymel-1* | 1.73 | 0.0031 | Nucleoside-triphosphatase activity |
| *Mal-A3* | 1.82 | 0.0018 | Carbohydrate metabolic process | *Eif3-S10* | 1.61 | 0.0002 | Organic cyclic compound binding |
| *Man2B1* | 2.00 | 0.0030 | Carbohydrate metabolic process | *Etfa* | 1.66 | 0.0013 | Organic cyclic compound binding |
| *Bckdhb* | 1.96 | 0.0051 | Catalytic activity | *Rpii215* | 1.64 | 0.0180 | Organic cyclic compound binding |
| *Rabggtb* | 1.77 | 0.0005 | Catalytic activity | *Ccdc47* | 1.70 | 0.0030 | Organic substance metabolic process |
| *Bms1* | 2.02 | 0.0070 | Cellular component biogenesis | *Rtf1* | 1.72 | 0.0076 | Organic substance metabolic process |
| *Ercc1* | 1.69 | 0.0037 | Cellular component biogenesis | *Dhcr24* | 1.62 | 0.0030 | Oxidation-reduction proces |
| *Snu13* | 1.77 | 0.0085 | Cellular component biogenesis | *Impdh1B* | 1.69 | 0.0000 | Oxidation-reduction proces |
| *Dnajc2* | 2.27 | 0.0005 | Chromatin binding | *Plod3* | 1.61 | 0.0066 | Oxidation-reduction proces |
| *Pbrm1* | 1.91 | 0.0096 | Chromatin binding | *Pxt* | 1.67 | 0.0301 | Oxidation-reduction proces |
| *Cth* | 1.74 | 0.0029 | Cofactor binding | *Sccpdh* | 1.61 | 0.0063 | Oxidation-reduction proces |
| *Drice* | 1.86 | 0.0009 | Cysteine-type peptidase activity | *Ppn* | 1.78 | 0.0089 | Peptidase inhibitor activity |
| *Adrm1* | 1.61 | 0.0030 | Cytoplasm | *Tfpi* | 1.72 | 0.0273 | Peptidase inhibitor activity |
| *Epb41L1* | 2.03 | 0.0092 | Cytoskeletal protein binding | *Fas3* | 1.78 | 0.0040 | Protein binding |
| *Acta1* | 1.90 | 0.0048 | Developmental process | *Kpna6* | 1.66 | 0.0154 | Protein transporter activity |
| *Dsp1* | 2.06 | 0.0005 | DNA binding | *Plxdc2* | 1.87 | 0.0046 | Receptor activity |
| *Eif2S3* | 1.63 | 0.0001 | DNA helicase activity | *Depdc1B* | 1.66 | 0.0014 | Regulation of cellular proces |
| *Ruvbl2* | 1.66 | 0.0023 | DNA helicase activity | *Aael000794* | 1.70 | 0.0015 | Regulation of metabolic process |
| *Mcm2* | 1.67 | 0.0150 | DNA replication initiation | *Psmd2* | 1.75 | 0.0058 | Regulation of metabolic process |
| *Smc3* | 1.86 | 0.0259 | Ellular component biogenesis | *Mad* | 1.71 | 0.0114 | Response to endogenous stimulus |
| *Nup85* | 1.64 | 0.0071 | Envelope | *Alp-M* | 1.91 | 0.0019 | Response to stimulus |
| *Nop58* | 1.81 | 0.0010 | Enzyme regulator activity | *Aph-4* | 2.24 | 0.0034 | Response to stimulus |
| *Pcna* | 1.79 | 0.0043 | Enzyme regulator activity | *Ilf2* | 1.97 | 0.0109 | Response to stimulus |
| *Tws* | 1.98 | 0.0077 | Enzyme regulator activity | *Lig1* | 1.94 | 0.0356 | Response to stimulus |
| *Bicd* | 2.00 | 0.0030 | Establishment of localization | *Hsp83* | 1.62 | 0.0023 | Response to stress |
| *Cbp80* | 2.04 | 0.0189 | Establishment of localization | *Rbm4B* | 1.64 | 0.0009 | Ribonucleoprotein complex biogenesis |
| *Kpna2* | 1.92 | 0.0072 | Establishment of localization | *Rpl7A* | 1.63 | 0.0015 | Ribonucleoprotein complex biogenesis |
| *Nup155* | 1.73 | 0.0485 | Establishment of localization | *Rsl24D1* | 2.77 | 0.0035 | Ribonucleoprotein complex biogenesis |
| *Sec16A* | 1.86 | 0.0354 | Establishment of localization | *Mcm3* | 1.62 | 0.0198 | Ribonucleotide binding |
| *Tnpo1* | 1.79 | 0.0065 | Establishment of localization | *Fubp3* | 1.87 | 0.0004 | RNA binding |
| *Ap1G1* | 1.71 | 0.0018 | Establishment of protein localization | *Zetatry* | 1.94 | 0.0037 | Serine-type endopeptidase activity |
| *Oxa1L* | 1.68 | 0.0073 | Establishment of protein localization | *Eif3-S8* | 1.62 | 0.0008 | Signal transducer activity |
| *Cse1L* | 1.66 | 0.0061 | Establishment of protein localization | *Ost-1* | 1.84 | 0.0194 | Signal transduction |
| *Cav1* | 2.53 | 0.0012 | Golgi membrane | *Mrpl19* | 1.93 | 0.0048 | Structural constituent of ribosome |
| *Eftud2* | 1.67 | 0.0035 | GTP binding | *Mrpl22* | 1.65 | 0.0085 | Structural constituent of ribosome |
| *L(2)Tid* | 1.64 | 0.0019 | Heat shock protein binding | *Mrps30* | 1.65 | 0.0025 | Structural constituent of ribosome |
| *Acy1* | 1.71 | 0.0027 | Hydrolase activity | *Mrps9* | 1.64 | 0.0056 | Structural constituent of ribosome |
| *Amy1* | 1.64 | 0.0099 | Hydrolase activity | *Rpl17* | 1.65 | 0.0018 | Structural constituent of ribosome |
| *Cphe* | 1.76 | 0.0012 | Hydrolase activity | *Rpl23* | 1.70 | 0.0009 | Structural constituent of ribosome |
| *Eif4A2* | 1.62 | 0.0023 | Hydrolase activity | *Rpl35A* | 1.71 | 0.0114 | Structural constituent of ribosome |
| *Entpd5* | 1.88 | 0.0002 | Hydrolase activity | *Rpl36* | 1.61 | 0.0038 | Structural constituent of ribosome |
| *Trappc5* | 1.93 | 0.0068 | Hydrolase activity | *Rplp0* | 1.74 | 0.0240 | Structural constituent of ribosome |
| *Usp15* | 1.64 | 0.0065 | Hydrolase activity | *Rps16* | 1.80 | 0.0014 | Structural constituent of ribosome |
| *Ptges2* | 1.62 | 0.0012 | Isomerase activity | *Rps7* | 1.82 | 0.0083 | Structural constituent of ribosome |
| *Gnl2* | 1.98 | 0.0124 | Membrane-enclosed lumen | *Fbn2* | 1.98 | 0.0010 | Structural molecule activity |
| *Smyd5* | 1.69 | 0.0024 | Membrane-enclosed lumen | *Krt8* | 2.14 | 0.0005 | Structural molecule activity |
| *Cg4406* | 1.85 | 0.0062 | Metabolic process | *Lam* | 1.79 | 0.0019 | Structural molecule activity |
| *Nero* | 1.88 | 0.0027 | Metabolic process | *Setd3* | 1.81 | 0.0266 | Transcription coactivator activity |
| *Pus7* | 1.84 | 0.0001 | Metabolic process | *Mta1* | 2.70 | 0.0039 | Transcription factor activity |
| *Def8* | 1.63 | 0.0022 | Metal ion binding | *Pacsin1B* | 1.65 | 0.0003 | Transcription factor activity |
| *Parn* | 1.88 | 0.0018 | [Metal ion binding](http://amigo.geneontology.org/amigo/term/GO:0046872) | *Polr1C* | 1.69 | 0.0468 | Transferase activity |
| *Mars* | 1.68 | 0.0081 | Methionine-tRNA ligase activity | *Aael007945* | 1.97 | 0.0050 | Translation initiation factor activity |
| *Emg1* | 1.74 | 0.0205 | Methyltransferase activity | *Iars2* | 1.67 | 0.0044 | tRNA aminoacylation |
| *C1Qbp* | 1.61 | 0.0013 | Mitochondrial matrix | *Cul3* | 1.74 | 0.0032 | Ubiquitin protein ligase binding |
| *Ncapg* | 1.62 | 0.0123 | Mitotic chromosome condensation | *Pfdn2* | 1.99 | 0.0085 | Unfolded protein binding |
| *Bel* | 1.66 | 0.0007 | Nucleic acid binding | *Pfdn6* | 1.65 | 0.0038 | Unfolded protein binding |
| *Ddx17* | 2.25 | 0.0001 | Nucleic acid binding | *Wls* | 1.98 | 0.0017 | Wnt signaling pathway |
| *Ddx18* | 2.10 | 0.0087 | Nucleic acid binding | *Cbs* | 3.42 | 0.0070 | Zinc ion binding |
| *Elavl4* | 1.92 | 0.0028 | Nucleic acid binding | *Sf3A3* | 2.03 | 0.0290 | Zinc ion binding |
