# Supplementary material for: Transcriptional and Translational Relationship in Environmental Stress: RNAseq and ITRAQ Proteomic Analysis Between Sexually Reproducing and Parthenogenetic Females in Moina micrura
Source: Front Physiol. 2018 Jul 2;9:812. doi: 10.3389/fphys.2018.00812 (PMC6036137; doi:10.3389/fphys.2018.00812)
Supplement: Supplementary file 6 [file Table_6.DOCX]

**Supplemental Table S6**

**The top 40 up- and down-regulated genes in *Moina micruras* (SF vs. PF).**

| **Up-regulated** | | | **Down-regulated** | | |
| --- | --- | --- | --- | --- | --- |
| **Gene** | **FC^SF^/_PF_** | **FDR** | **Gene** | **FC^PF^/_SF_** | **FDR** |
| *N/A^d^* | 51399.00 | 7.65E-14 | *Fxr1* | 165594.50 | 1.38E-20 |
| *N/A^e^* | 44274.80 | 1.61E-19 | *Tmem63A* | 102280.00 | 5.50E-16 |
| *Lgals6* | 15666.67 | 1.64E-13 | *Resilin* | 38732.54 | 3.02E-22 |
| *Cryaa* | 8091.64 | 5.40E-27 | *Bap60* | 7824.75 | 4.22E-14 |
| *H4* | 7878.83 | 1.53E-13 | *Zgc:66014* | 6058.33 | 1.14E-14 |
| *Stxbp5* | 5500.00 | 1.63E-09 | *Etfa* | 4877.20 | 1.05E-20 |
| *Vat1L* | 4971.83 | 3.78E-26 | *Mlc1* | 2946.46 | 1.07E-22 |
| *Edc3* | 3319.00 | 9.99E-09 | *Tsr* | 2531.81 | 1.95E-19 |
| Hemoglobin | 3030.29 | 2.74E-21 | *Odc1* | 1960.68 | 1.56E-18 |
| *Gpx5* | 2756.00 | 2.23E-20 | *Mfsd11* | 1617.82 | 9.18E-18 |
| *Tdpoz5* | 2640.00 | 3.31E-20 | *Nsun2* | 1450.69 | 2.42E-17 |
| *Ref(2)P* | 2515.00 | 5.24E-20 | *Mmd2* | 1422.52 | 2.85E-17 |
| *H2a.F/Z* | 2058.81 | 3.04E-19 | *Cfap20* | 1377.16 | 9.20E-11 |
| *N/A^c^* | 2034.15 | 1.66E-23 | *Gpx5* | 1079.00 | 3.60E-16 |
| *Taf5L* | 1910.27 | 6.15E-19 | *Pgant9* | 912.25 | 1.51E-15 |
| *Hsd17B12* | 1879.33 | 6.24E-21 | *Pigm* | 698.00 | 1.63E-14 |
| *Mettl10* | 1759.13 | 7.40E-23 | *Cg4928* | 660.27 | 2.65E-14 |
| *Zgc:66014* | 1363.63 | 1.33E-14 | *Atoh8* | 644.79 | 2.21E-09 |
| *Znfx1* | 1304.61 | 1.99E-17 | *Gas2* | 634.81 | 3.84E-14 |
| *Tpsb* | 1235.42 | 1.75E-16 | *Pfk* | 629.48 | 8.58E-11 |
| *Hsp-16.2* | 1074.67 | 8.76E-19 | *Ebp2* | 599.72 | 6.13E-14 |
| *Sdha* | 950.85 | 3.50E-16 | *Plekhf2* | 580.02 | 8.17E-14 |
| *Mgst3* | 925.11 | 4.32E-16 | *Aael007945* | 511.58 | 2.38E-13 |
| *Tmem63A* | 905.71 | 5.17E-16 | *Lgals6* | 506.97 | 2.59E-13 |
| *Prss1* | 903.90 | 5.22E-16 | *Atpalpha* | 480.72 | 4.23E-13 |
| *Rtf1* | 898.25 | 5.50E-16 | *Rgd1559896* | 479.87 | 4.30E-13 |
| *Mcm4* | 884.40 | 6.36E-16 | *Pcgf3* | 439.40 | 9.61E-13 |
| *Serpinb10* | 825.01 | 1.18E-15 | *Hop3* | 381.63 | 3.43E-12 |
| *Acsbg2* | 809.82 | 1.39E-15 | *Gabpa* | 371.85 | 4.29E-12 |
| *Bmpr2* | 799.28 | 1.55E-15 | *Cg8135* | 369.22 | 4.64E-12 |
| *Vg2* | 793.82 | 1.61E-20 | *Gk* | 359.07 | 5.81E-12 |
| *Mthl5* | 737.77 | 3.24E-15 | *Rab40C* | 342.49 | 8.96E-12 |
| *Alx-1* | 737.43 | 8.60E-18 | *Svep1* | 335.44 | 1.09E-11 |
| *Psme3* | 734.54 | 3.34E-15 | *Dync1Li1* | 333.65 | 1.12E-11 |
| *Mab-21* | 727.64 | 3.58E-15 | *Dusp10* | 328.86 | 1.25E-11 |
| *Uqcrb* | 670.52 | 7.36E-15 | *Loc411229* | 327.18 | 1.32E-11 |
| *Atp5S* | 660.89 | 8.50E-15 | *Gpkow* | 308.62 | 2.21E-11 |
| *Ubxn4* | 655.02 | 9.10E-15 | *Sars* | 307.24 | 2.35E-11 |
| *Cht3* | 602.36 | 1.94E-14 | *Aspm* | 296.87 | 3.12E-11 |
| *Pigm* | 598.00 | 2.04E-14 | *Clc* | 287.29 | 4.33E-11 |

**Note***: N/A^c^*: Cuticle protein 7; *N/A^d^*: Venom dipeptidyl peptidase 4; *N/A^e^*: Histone H1-delta
