# Supplementary material for: Transcriptional and Translational Relationship in Environmental Stress: RNAseq and ITRAQ Proteomic Analysis Between Sexually Reproducing and Parthenogenetic Females in Moina micrura
Source: Front Physiol. 2018 Jul 2;9:812. doi: 10.3389/fphys.2018.00812 (PMC6036137; doi:10.3389/fphys.2018.00812)
Supplement: Supplementary file 7 [file Table_7.DOCX]

**Supplemental Table S7**

**The top 40 up- and down-regulated proteins** **in *Moina micruras* (SF vs. PF).**

| **Up-regulated** | | | **Down-regulated** | | |
| --- | --- | --- | --- | --- | --- |
| **Gene** | **FC^SF^/_PF_** | **P-value** | **Gene** | **FC^PF^/_SF_** | **P-value** |
| Hemoglobin | 5.63 | 0.0001574 | *Npc2a* | 4.71 | 0.0012494 |
| *Sod* | 3.78 | 0.0028876 | *Clca4a* | 3.88 | 0.0005518 |
| *Vg2* | 3.68 | 0.0034204 | *Cbs* | 3.42 | 0.0069758 |
| *Vg* | 3.55 | 0.0020473 | *Npc2* | 3.39 | 0.0012802 |
| *SodF* | 3.33 | 0.0001553 | *Heh-1* | 3.26 | 0.0004079 |
| *Vg2* | 3.04 | 0.0024543 | *Noc2l* | 3.01 | 0.0117866 |
| *N/A^f^* | 2.91 | 0.0074098 | *N/A^h^* | 2.95 | 0.0004632 |
| *N/A^g^* | 2.61 | 0.0065613 | *N/A^j^* | 2.90 | 0.0012005 |
| *Sls* | 2.59 | 0.0008662 | *Med21* | 2.81 | 0.0094518 |
| *Smtnl1* | 2.57 | 0.0021047 | Hemoglobin | 2.81 | 0.0002181 |
| *Rpl27c* | 2.55 | 0.0063470 | *Rsl24d1* | 2.77 | 0.0035052 |
| *Tpd52* | 2.32 | 0.0119145 | *Mta1* | 2.70 | 0.0039225 |
| *Mettl10* | 2.28 | 0.0006809 | *Histone H3* | 2.63 | 0.0047861 |
| *Ga18377* | 2.26 | 0.0076219 | *Cav1* | 2.53 | 0.0012062 |
| *Ferh* | 2.25 | 0.0060266 | *Btf3* | 2.48 | 0.0021748 |
| *Ddb_g0274169* | 2.17 | 0.0018819 | *Cg8005* | 2.47 | 0.0024684 |
| *Vat1l* | 2.15 | 0.0068382 | *Ddx27* | 2.47 | 0.0009806 |
| *Apod* | 2.13 | 0.0061593 | *Coprox* | 2.42 | 0.0029287 |
| *Cryaa* | 2.12 | 0.0086514 | *Pcbp2* | 2.42 | 0.0014839 |
| *Hsp-16.2* | 2.11 | 0.0085331 | *N/A^i^* | 2.39 | 0.0012566 |
| *Cat-1* | 2.01 | 0.0113644 | *Tle4* | 2.38 | 0.0061668 |
| *Htb1* | 2.00 | 0.0083126 | *W* | 2.38 | 0.0007670 |
| *Mp20* | 1.97 | 0.0002104 | *Nup160* | 2.34 | 0.0007682 |
| *Amy2* | 1.97 | 0.0024394 | *Mettl7b* | 2.34 | 0.0193906 |
| *Rps26* | 1.95 | 0.0018407 | *Mrps27* | 2.30 | 0.0079544 |
| *Osj_11358* | 1.94 | 0.0061628 | *Chp1* | 2.28 | 0.0059075 |
| *Ap2s1* | 1.92 | 0.0013912 | *Csnk2a1* | 2.28 | 0.0008222 |
| *N/A^a^* | 1.91 | 0.0113332 | *Dnajc2* | 2.27 | 0.0005420 |
| *Cg2145* | 1.87 | 0.0003961 | *Cdo1* | 2.25 | 0.0104768 |
| *Ndufaf5* | 1.87 | 0.0076539 | *Ddx17* | 2.25 | 0.0001055 |
| *Pxt* | 1.85 | 0.0023514 | *Aph-4* | 2.24 | 0.0033782 |
| *N/A^c^* | 1.83 | 0.0007276 | *Fax* | 2.21 | 0.0003310 |
| *Cygb2* | 1.82 | 0.0101651 | *Cut-1* | 2.20 | 0.0029262 |
| *Cpa2* | 1.80 | 0.0040518 | *Fam192a* | 2.19 | 0.0233470 |
| *Ndufb3* | 1.79 | 0.0069295 | *N/A^k^* | 2.16 | 0.0016220 |
| *Rps14a* | 1.78 | 0.0019982 | *Nacalpha* | 2.16 | 0.0002231 |
| *Gvin1* | 1.77 | 0.0462889 | *Sec5* | 2.15 | 0.0057749 |
| *Rpl23p* | 1.74 | 0.0057569 | *Krt8* | 2.14 | 0.0004588 |
| *Gpx3* | 1.73 | 0.0272180 | *Strip1* | 2.13 | 0.0011749 |
| *Slc6a1* | 1.73 | 0.0185625 | *Mki67* | 2.12 | 0.0003731 |

**Note**: *N/A^a^*: Endocuticle structural glycoprotein SgAbd-8; *N/A^c^*: Cuticle protein 7; *N/A^f^*: V-type proton ATPase subunit G; *N/A^g^*: Troponin C; *N/A^i^*: Endocuticle structural glycoprotein SgAbd-1; *N/A^h^*: Endocuticle structural glycoprotein SgAbd-3; *N/A^j^:* mRNA-capping enzyme; *N/A^k^*: MAM and LDL-receptor class A domain-containing protein 2
