# Supplementary material for: Transcriptional and Translational Relationship in Environmental Stress: RNAseq and ITRAQ Proteomic Analysis Between Sexually Reproducing and Parthenogenetic Females in Moina micrura
Source: Front Physiol. 2018 Jul 2;9:812. doi: 10.3389/fphys.2018.00812 (PMC6036137; doi:10.3389/fphys.2018.00812)
Supplement: Supplementary file 8 [file Table_8.DOCX]

**Supplemental Table S8**

**Reproductive switching most-related proteins in *Moina micruras* (SF vs. PF).**

| **Protein** | **P-value** | **FC^SF^/_SP_** | **Gene** | **FC^SF^/_SP_** | **FDR** |
| --- | --- | --- | --- | --- | --- |
| Vitellogenin-2 | 0.003420 | 3.68 | *Vg2* | 793.82 | 1.61E-20 |
| EEF1A lysine methyltransferase 2 | 0.000681 | 2.28 | *Mettl10* | 1759.13 | 7.40E-23 |
| Synaptic vesicle membrane protein VAT-1 homolog-like | 0.006838 | 2.15 | *Vat1L* | 4971.83 | 1.87E-30 |
| Alpha-crystallin A chain | 0.008651 | 2.12 | *Cryaa* | 8091.64 | 5.40E-27 |
| Heat shock protein Hsp-16.2 | 0.008533 | 2.11 | *Hsp-16.2* | 1074.67 | 8.76E-19 |
