# Supplementary material for: Transcriptional and Translational Relationship in Environmental Stress: RNAseq and ITRAQ Proteomic Analysis Between Sexually Reproducing and Parthenogenetic Females in Moina micrura
Source: Front Physiol. 2018 Jul 2;9:812. doi: 10.3389/fphys.2018.00812 (PMC6036137; doi:10.3389/fphys.2018.00812)
Supplement: Supplementary file 9 [file Table_9.DOCX]

**Supplemental Table S9**

**The protein of** **significantly up-regulated at the protein level and insignificantly up-regulated at the genes level in *Moina micruras* (SF vs. PF).**

| **Protein** | **FC^SF^/_PF_** | **P-value** | **Gene** | **FC^SF^/_PF_** | **FDR** |
| --- | --- | --- | --- | --- | --- |
| Hemoglobin | 5.63 | 0.0001574 | Hemoglobin | 23.08 | 2.74E-21 |
| Superoxide dismutase [Cu-Zn] | 3.78 | 0.0028876 | *Sod* | 15.68 | 2.52E-08 |
| Vitellogenin-2 | 3.04 | 0.0024543 | *Vg2* | 26.47 | 1.61E-20 |
| Pancreatic alpha-amylase | 1.97 | 0.0024394 | *Amy2* | 23.95 | 4.46E-10 |
| 40S ribosomal protein S26 | 1.95 | 0.0018407 | *Rps26* | 7.83 | 1.31E-05 |
