# Supplementary material for: Transcriptional and Translational Relationship in Environmental Stress: RNAseq and ITRAQ Proteomic Analysis Between Sexually Reproducing and Parthenogenetic Females in Moina micrura
Source: Front Physiol. 2018 Jul 2;9:812. doi: 10.3389/fphys.2018.00812 (PMC6036137; doi:10.3389/fphys.2018.00812)
Supplement: Supplementary file 10 [file Table_10.DOCX]

**Supplemental Table S10**

**The protein of significantly down-regulated at the protein level and insignificantly down-regulated at the genes level in *Moina micruras* (SF vs. PF).**

| **Protein** | **FC^PF^/_SF_** | **P-value** | **Gene** | **FC^PF^/_SF_** | **FDR** |
| --- | --- | --- | --- | --- | --- |
| Calcium-activated chloride channel regulator 4A | 3.88 | 0.0005518 | *Clca4a* | 9.37 | 0.000143 |
| Epididymal secretory protein E1 | 3.39 | 0.0012802 | *Npc2* | 8.48 | 4.12E-05 |
| Endocuticle structural glycoprotein SgAbd-1 | 2.39 | 0.0012566 | *N/A^i^* | 4.96 | 0.001828 |
| Exocyst complex component 2 | 2.15 | 0.0057749 | *Sec5* | 12.84 | 2.06E-06 |
| Keratin, type II cytoskeletal 8 | 2.14 | 0.0004588 | *Krt8* | 14.00 | 0.002864 |
| UDP-glucuronosyltransferase 2B31 | 2.12 | 0.0028458 | *Ugt2b31* | 25.52 | 3.10E-08 |
| Lysosomal alpha-mannosidase | 2.00 | 0.0030018 | *Man2b1* | 28.17 | 6.06E-09 |

*N/A^i^*: Endocuticle structural glycoprotein SgAbd-1
