# Supplementary material for: Transcriptional and Translational Relationship in Environmental Stress: RNAseq and ITRAQ Proteomic Analysis Between Sexually Reproducing and Parthenogenetic Females in Moina micrura
Source: Front Physiol. 2018 Jul 2;9:812. doi: 10.3389/fphys.2018.00812 (PMC6036137; doi:10.3389/fphys.2018.00812)
Supplement: Supplementary file 11 [file Table_11.DOCX]

**Supplemental Table S11**

**The protein of significantly up-regulated at the protein level and no differentially expressed at the genes level in *Moina micruras* (SF vs. PF).**

| **Protein** | **FC(^SF^/_PF_)** | **P-value** | **Gene** | **FC(^SF^/_PF_)** | **FDR** |
| --- | --- | --- | --- | --- | --- |
| Vitellogenin fused with superoxide dismutase | 3.33 | 0.0001553 | *Dmagvtg1* | 1.04 | 1.0000000 |
| V-type proton ATPase subunit G | 2.91 | 0.0074098 | *-* | 0.46 | 0.4535851 |
| Troponin C | 2.61 | 0.0065613 | *Tnc1* | 0.25 | 0.0805200 |
| Titin | 2.59 | 0.0008662 | *Sls* | 0.64 | 0.7253701 |
| Smoothelin-like protein 1 | 2.57 | 0.0021047 | *Smtnl1* | 0.46 | 0.4596620 |
| 60S ribosomal protein L27-3 | 2.55 | 0.0063470 | *Rpl27c* | 0.43 | 0.7253701 |
| Tumor protein D52 | 2.32 | 0.0119145 | *Tpd52* | 0.66 | 0.7293213 |
| Coiled-coil and C2 domain-containing protein 1-like | 2.26 | 0.0076219 | *Ga18377* | 1.04 | 0.8805581 |
| Ferritin subunit | 2.25 | 0.0060266 | *Ferh* | 0.13 | 0.0087660 |
| Cathepsin L-like proteinase | 2.01 | 0.0113644 | *Cat-1* | 1.91 | 0.3457043 |
| Histone H2B OS=Encephalitozoon cuniculi (strain GB-M1) | 2.00 | 0.0083126 | *Htb1* | 0.91 | 1.0000000 |
| Cysteine proteinase inhibitor 8 | 1.94 | 0.0061628 | *Os03g0429000* | 1.43 | 0.5740710 |
| AP-2 complex subunit sigma | 1.92 | 0.0013912 | *Ap2s1* | 0.45 | 0.4535851 |
| NADH dehydrogenase 1 alpha subcomplex assembly factor | 1.87 | 0.0076539 | *Ndufaf5* | 0.79 | 0.9444270 |
| Chorion peroxidase | 1.85 | 0.0023514 | *Pxt* | 0.59 | 0.7086529 |
| Cuticle protein 7 | 1.83 | 0.0007276 | *-* | 3.03 | 0.0821342 |
| Cytoglobin-2 | 1.82 | 0.0101651 | *Cygb2* | 0.96 | 0.9646688 |
| Carboxypeptidase A2 | 1.80 | 0.0040518 | *Cpa2* | 0.18 | 0.0150361 |
| NADH dehydrogenase 1 beta subcomplex subunit 3 | 1.79 | 0.0069295 | *Ndufb3* | 14.65 | 0.0180803 |
| 40S ribosomal protein S14 | 1.78 | 0.0019982 | *Rps14a* | 0.88 | 1.0000000 |
| Interferon-induced very large GTPase 1 | 1.77 | 0.0462889 | *Gvin1* | 0.95 | 0.9763439 |
| 50S ribosomal protein L23P | 1.74 | 0.0057569 | *Rpl23p* | 1.50 | 0.9337285 |
| Sodium- and chloride-dependent GABA transporter 1 | 1.73 | 0.0185625 | *Slc6a1* | 0.28 | 0.1171510 |
| Branched-chain-amino-acid aminotransferase, cytosolic | 1.71 | 0.0023914 | *Bcat1* | 1.07 | 0.8421225 |
| Probable ATP-dependent RNA helicase spindle-E | 1.68 | 0.0015801 | *Spn-E* | 2.26 | 0.2276769 |
| Membrane metallo-endopeptidase-like 1 | 1.68 | 0.0001923 | *Mmel1* | 0.20 | 0.0403721 |
| Myosin light chain alkali | 1.67 | 0.0203734 | *Mlc1* | 0.51 | 0.5712031 |
| Endocuticle structural glycoprotein SgAbd-2 | 1.65 | 0.0239360 | *-* | 0.00 | 1.0000000 |
| Protein-glutamate O-methyltransferase | 1.64 | 0.0000945 | *Armt1* | 1.57 | 0.5277421 |
| 60S ribosomal protein L15 | 1.64 | 0.0212960 | *Rpl15* | 0.55 | 0.7796751 |
| Hemocyte protein-glutamine gamma-glutamyltransferase | 1.64 | 0.0003710 | *-* | 1.90 | 0.3457043 |
| Transmembrane protein 222 | 1.63 | 0.0166291 | *Tmem222* | 2.66 | 0.1342786 |
| Elongation factor 1-alpha | 1.62 | 0.0025691 | *Tef1* | 0.88 | 1.0000000 |
| Neurogenic locus notch homolog protein 3 | 1.62 | 0.0087545 | *Notch3* | 1.59 | 0.5034644 |
| Probable cytochrome P450 301a1, mitochondrial | 1.61 | 0.0073307 | *Cyp301a1* | 1.03 | 0.8814535 |
| Dual specificity protein phosphatase 3 | 1.60 | 0.0085684 | *Dusp3* | 0.80 | 0.9687427 |
| Pupal cuticle protein 20 | 1.60 | 0.0113674 | *Pcp20* | 0.64 | 0.7253701 |
| Calcyphosin-like protein | 1.59 | 0.0024624 | *Capsl* | 0.61 | 0.7253701 |
| Heat shock protein ECU02_0100 | 1.59 | 0.0025001 | *Ecu02_0100* | 0.35 | 0.4072912 |
| Kynurenine formamidase | 1.58 | 0.0145416 | *Kynb* | 3.44 | 0.0558896 |
| Muscle-specific protein 20 | 1.58 | 0.0013342 | *Mp20* | 0.42 | 0.3907287 |
| Ferritin heavy chain | 1.56 | 0.0068386 | *-* | 0.12 | 0.0031195 |
| 2,4-dienoyl-CoA reductase, mitochondrial | 1.54 | 0.0223232 | *Decr1* | 0.87 | 1.0000000 |
| Venom carboxylesterase-6 | 1.53 | 0.0139129 | *-* | 0.81 | 0.9724876 |
| Adenine phosphoribosyltransferase | 1.53 | 0.0020135 | *Apt1* | 0.99 | 0.9305927 |
| Multiple inositol polyphosphate phosphatase 1 | 1.52 | 0.0482272 | *Minpp1* | 0.86 | 1.0000000 |
| Short-chain dehydrogenase/reductase family 16C member 6 | 1.52 | 0.0044153 | *Sdr16c6* | 1.16 | 0.7928939 |
| Carboxypeptidase A2 | 1.51 | 0.0023318 | *Cpa2* | 0.68 | 0.7757389 |
