# Supplementary material for: Transcriptional and Translational Relationship in Environmental Stress: RNAseq and ITRAQ Proteomic Analysis Between Sexually Reproducing and Parthenogenetic Females in Moina micrura
Source: Front Physiol. 2018 Jul 2;9:812. doi: 10.3389/fphys.2018.00812 (PMC6036137; doi:10.3389/fphys.2018.00812)
Supplement: Supplementary file 12 [file Table_12.DOCX]

**Supplemental Table S12**

**The protein of significantly down-regulated at the protein level and no differentially expressed at the genes level in *Moina micruras* (SF vs. PF).**

| **Protein** | **FC(^PF^/_SF_)** | **P-value** | **Gene** | **FC(^PF^/_SF_)** | **FDR** |
| --- | --- | --- | --- | --- | --- |
| Protein NPC2 homolog | 4.71 | 0.0012494 | *Npc2a* | 2.37 | 0.3868925 |
| Calcium-activated chloride channel regulator 4A | 3.88 | 0.0005518 | *Clca4a* | 9.37 | 0.0038910 |
| Cystathionine beta-synthase | 3.42 | 0.0069758 | *Cbs* | 0.69 | 0.5712031 |
| Epididymal secretory protein E1 | 3.39 | 0.0012802 | *Npc2* | 8.48 | 0.0014171 |
| Nucleolar complex protein 2 homolog | 3.01 | 0.0117866 | *Noc2l* | 0.77 | 0.7051755 |
| Endocuticle structural glycoprotein SgAbd-3 | 2.95 | 0.0004632 | *-* | 1.89 | 0.5712031 |
| mRNA-capping enzyme | 2.90 | 0.0012005 | *Rngtt* | 0.33 | 0.0799055 |
| Mediator of RNA polymerase II transcription subunit 21 | 2.81 | 0.0094518 | *Med21* | 1.15 | 1.0000000 |
| Globin | 2.81 | 0.0002181 | *-* | 0.60 | 0.4641571 |
| Probable ribosome biogenesis protein RLP24 | 2.77 | 0.0035052 | *Rsl24d1* | 0.75 | 0.7060773 |
| Metastasis-associated protein MTA1 | 2.70 | 0.0039225 | *Mta1* | 0.66 | 0.5712031 |
| Histone H3 | 2.63 | 0.0047861 | *-* | 0.74 | 0.6875895 |
| Caveolin-1 | 2.53 | 0.0012062 | *Cav1* | 2.52 | 0.3457043 |
| Transcription factor BTF3 | 2.48 | 0.0021748 | *Btf3* | 0.99 | 0.9077897 |
| Probable deoxyhypusine synthase | 2.47 | 0.0024684 | *Cg8005* | 0.81 | 0.7253701 |
| Probable ATP-dependent RNA helicase DDX27 | 2.47 | 0.0009806 | *Ddx27* | 0.75 | 0.6808671 |
| Oxygen-dependent coproporphyrinogen-III oxidase | 2.42 | 0.0029287 | *Coprox* | 0.47 | 0.3486322 |
| Poly(rC)-binding protein 2 | 2.42 | 0.0014839 | *Pcbp2* | 1.05 | 0.9744459 |
| Endocuticle structural glycoprotein SgAbd-1 | 2.39 | 0.0012566 | *-* | 4.96 | 0.0288866 |
| Transducin-like enhancer protein 4 | 2.38 | 0.0061668 | *Tle4* | 0.84 | 0.7253701 |
| Protein white | 2.38 | 0.0007670 | *W* | 0.81 | 0.7253701 |
| Nuclear pore complex protein Nup160 homolog | 2.34 | 0.0007682 | *Nup160* | 0.65 | 0.5712031 |
| Methyltransferase-like protein 7B | 2.34 | 0.0193906 | *Mettl7b* | 1.30 | 1.0000000 |
| 28S ribosomal protein S27, mitochondrial | 2.30 | 0.0079544 | *Mrps27* | 1.36 | 0.8663179 |
| Calcineurin B homologous protein 1 | 2.28 | 0.0059075 | *Chp1* | 1.28 | 0.9305792 |
| Casein kinase II subunit alpha | 2.28 | 0.0008222 | *Csnk2a1* | 1.05 | 0.9724876 |
| DnaJ homolog subfamily C member 2 | 2.27 | 0.0005420 | *Dnajc2* | 0.82 | 0.7253701 |
| Cysteine dioxygenase type 1 | 2.25 | 0.0104768 | *Cdo1* | 1.70 | 0.6952564 |
| Probable ATP-dependent RNA helicase DDX17 | 2.25 | 0.0001055 | *Ddx17* | 1.06 | 0.9899972 |
| Alkaline phosphatase 4 | 2.24 | 0.0033782 | *Aph-4* | 1.74 | 0.6730580 |
| Failed axon connections | 2.21 | 0.0003310 | *Fax* | 1.19 | 1.0000000 |
| Cuticlin-1 | 2.20 | 0.0029262 | *Cut-1* | 1.42 | 0.8162624 |
| Protein FAM192A | 2.19 | 0.0233470 | *-* | 0.95 | 0.8585935 |
| MAM and LDL-receptor class A domain-containing protein 2 | 2.16 | 0.0016220 | *-* | 0.42 | 0.1972555 |
| Nascent polypeptide-associated complex subunit alpha | 2.16 | 0.0002231 | *Nacalpha* | 1.09 | 1.0000000 |
| Keratin, type II cytoskeletal 8 | 2.14 | 0.0004588 | *Krt8* | 14.00 | 0.0391118 |
| Striatin-interacting protein 1 homolog | 2.13 | 0.0011749 | *Strip1* | 1.05 | 0.9807809 |
| Antigen KI-67 | 2.12 | 0.0003731 | *Mki67* | 0.76 | 0.6899491 |
| Nuclear receptor coactivator 5 | 2.12 | 0.0040442 | *Ncoa5* | 0.21 | 0.0290914 |
| 28S ribosomal protein S22, mitochondrial | 2.11 | 0.0119345 | *Mrps22* | 2.82 | 0.2666240 |
| Thioredoxin-related transmembrane protein 1 | 2.10 | 0.0071066 | *Tmx1* | 0.63 | 0.5159749 |
| ATP-dependent RNA helicase DDX18 | 2.10 | 0.0087417 | *Ddx18* | 0.85 | 0.7253701 |
| DNA-directed RNA polymerase II subunit RPB4 | 2.09 | 0.0145161 | *Polr2d* | 0.07 | 0.0337903 |
| Pre-mRNA-splicing factor CWC22 homolog | 2.09 | 0.0029924 | *Ncm* | 1.08 | 1.0000000 |
| Histone H2A | 2.08 | 0.0023430 | *-* | 0.40 | 0.1643529 |
| Nucleolar transcription factor 1 | 2.08 | 0.0339211 | *Ubtf* | 0.69 | 0.5712031 |
| Acyl-CoA synthetase family member 4 | 2.08 | 0.0038400 | *Aasdh* | 1.21 | 1.0000000 |
| Carboxypeptidase B | 2.08 | 0.0045026 | *-* | 8.17 | 0.0016570 |
| Nuclear pore complex protein Nup98-Nup96 | 2.08 | 0.0008506 | *Nup98* | 0.45 | 0.2469473 |
| Globin C, coelomic | 2.08 | 0.0012692 | *-* | 2.70 | 0.2910337 |
| High mobility group protein DSP1 | 2.06 | 0.0004785 | *Dsp1* | 0.50 | 0.4535851 |
| Fatty acid-binding protein, muscle | 2.06 | 0.0015174 | *-* | 0.24 | 0.1627584 |
| Zinc finger CCCH domain-containing protein 15 homolog | 2.05 | 0.0041292 | *Ga21225* | 0.90 | 0.8006494 |
| Glycosyltransferase-like domain-containing protein 1-like | 2.05 | 0.0015262 | *Cg15914* | 0.53 | 0.3849509 |
| Nuclear cap-binding protein subunit 1 | 2.04 | 0.0189074 | *Cbp80* | 0.61 | 0.4916682 |
| Band 4.1-like protein 1 | 2.03 | 0.0092345 | *Epb41l1* | 1.23 | 0.9818986 |
| Methionine aminopeptidase 2 | 2.03 | 0.0000362 | *Metap2* | 0.89 | 0.7803676 |
| Splicing factor 3A subunit 3 | 2.03 | 0.0290095 | *Sf3a3* | 0.64 | 0.5366213 |
| Ribosome biogenesis protein BMS1 homolog | 2.02 | 0.0070155 | *Bms1* | 0.93 | 0.8327831 |
| Tubulin alpha-1 chain | 2.02 | 0.0025923 | *Alphatub84b* | 1.02 | 0.9396472 |
| Insulin-like growth factor 2 mRNA-binding protein 1 | 2.02 | 0.0044065 | *Igf2bp1* | 1.29 | 0.9261850 |
| La-related protein CG11505 | 2.01 | 0.0027732 | *Cg11505* | 0.99 | 0.8993751 |
| Protein bicaudal D | 2.00 | 0.0030034 | *Bicd* | 1.05 | 0.9724876 |
| Prefoldin subunit 2 | 1.99 | 0.0084564 | *Pfdn2* | 0.97 | 0.8920197 |
| Protein phosphatase PP2A 55 kDa regulatory subunit | 1.98 | 0.0076731 | *Tws* | 1.25 | 0.9657471 |
