# Supplementary material for: Transcriptional and Translational Relationship in Environmental Stress: RNAseq and ITRAQ Proteomic Analysis Between Sexually Reproducing and Parthenogenetic Females in Moina micrura
Source: Front Physiol. 2018 Jul 2;9:812. doi: 10.3389/fphys.2018.00812 (PMC6036137; doi:10.3389/fphys.2018.00812)
Supplement: Supplementary file 13 [file Table_13.DOCX]

**Supplemental Table S13**

**The protein of significantly up-regulated at the protein level and opposite expression at the genes level in *Moina micruras* (SF vs. PF).**

| **Protein** | **FC^SF^/_PF_** | **P-value** | **Gene** | **FC^PF^/_SF_** | **FDR** |
| --- | --- | --- | --- | --- | --- |
| Poly(U)-specific endoribonuclease homolog | 1.87 | 0.0003961 | *Cg2145* | 9.07 | 0.0008957 |
| - | 1.57 | 0.0061056 | - | 9.09 | 0.0008161 |
| Apolipoprotein D | 2.13 | 0.0061593 | *Apod* | 11.00 | 0.0002418 |
| Oplophorus-luciferin 2-monooxygenase non-catalytic subunit | 1.54 | 0.0013170 | - | 13.99 | 6.84E-05 |
