# Supplementary material for: Transcriptional and Translational Relationship in Environmental Stress: RNAseq and ITRAQ Proteomic Analysis Between Sexually Reproducing and Parthenogenetic Females in Moina micrura
Source: Front Physiol. 2018 Jul 2;9:812. doi: 10.3389/fphys.2018.00812 (PMC6036137; doi:10.3389/fphys.2018.00812)
Supplement: Supplementary file 14 [file Table_14.DOCX]

**Supplemental Table S14**

**The protein of significantly down-regulated at the protein level and opposite expression at the genes level in *Moina micruras* (SF vs. PF).**

| **Protein** | **FC^PF^/_SF_** | **P-value** | **Gene** | **FCs^SF^/_PF_** | **FDR** |
| --- | --- | --- | --- | --- | --- |
| Transforming growth factor-beta-induced protein ig-h3 | 1.59 | 0.000887222 | *Tgfbi* | 27.98 | 0.0008388 |
| Ubiquitin fusion degradation protein 1 homolog | 1.64 | 0.006619462 | *Ufd1l* | 27.77 | 0.0008388 |
| Transcription initiation factor IIB | 1.66 | 0.012969550 | *Tfiib* | 8.23 | 0.0007534 |
| tRNA (cytosine(34)-C(5))-methyltransferase | 1.57 | 0.016718674 | *Nsun2* | 26.31 | 4.40E-08 |
| Meiotic nuclear division protein 1 homolog | 1.62 | 0.042782103 | *Mnd1* | 199.56 | 3.29E-10 |
| Putative protein heh-1 | 1.32 | 0.000407914 | *Heh-1* | 137.34 | 1.24E-12 |
| Fragile X mental retardation syndrome-related protein 1 | 1.64 | 0.013576472 | *Fmr1* | 126.52 | 9.20E-14 |
| - | 1.34 | 0.000123513 | *-* | 493.29 | 4.08E-16 |
| - | 1.42 | 0.005244773 | *-* | 1174.72 | 1.56E-16 |
| Thymosin beta-11 | 1.53 | 0.001594157 | *Tyb11* | 1174.72 | 1.18E-16 |
| Histone H1-delta | 1.63 | 0.010873888 | *H1d* | 1924.99 | 1.61E-19 |
