# Supplementary material for: Transcriptional and Translational Relationship in Environmental Stress: RNAseq and ITRAQ Proteomic Analysis Between Sexually Reproducing and Parthenogenetic Females in Moina micrura
Source: Front Physiol. 2018 Jul 2;9:812. doi: 10.3389/fphys.2018.00812 (PMC6036137; doi:10.3389/fphys.2018.00812)
Supplement: Supplementary file 15 [file Presentation_1.PDF]

**Supplemental Information for Methods**

# Transcriptional and translational relationship in environmental stress: RNAseq and ITRAQ proteomic analysis between sexually reproducing and parthenogenetic females in *Moina micrura*

Jingyi Jia#, Xiangjiang Liu#, Lu Li, Ying Dong, Chengqiang Lei, Guoqiang Wu, Guangfu Hu

College of Fisheries, Huazhong Agricultural University, Wuhan, China, 430070

# Both Jingyi Jia and Xiangjiang Liu contributed equally to this work.

**Key words:** *Moina micrura*, sexual female, parthenogenetic female, RNA-Seq, iTRAQ

**Abbreviations:** PF, parthenogenetic female; SF, sexual female; iTRAQ, isobaric tag for relative absolute quantitation; Vg, vitellogenin; DEGs, differential expressed genes; DEPs, differential expressed proteins;

**No. of Figures:** 6 figures and 5 tables (plus 14 supplemental tables)

**Disclosure statement:** The authors have nothing to disclose for potential conflict of interest.

**Please address correspondence and reprint request to:**

Dr. Xiangjiang Liu

College of Fisheries, Huazhong Agricultural University, Wuhan, 430070, China

(Phone: 86-15827302886, Email: liuxiangjiang@mail.hzau.edu.cn )

## Materials and methods

### Protein preparation and iTRAQ labeling.

Remove the quick-frozen sample and add the appropriate amount of lysis buffer (8 M urea, 0.3% SDS) and Protease Inhibitor (Thermo, USA) directly and sonicated in ice. The protein (200 µg in each sample, increasing the volume to 200µL with lysate.) was reduced with 10 mM TCEP (Thermo, USA) (final concentration) at 37 °C for 1 h and alkylated by 40 mM iodoacetamide (Sigma, USA) in the darkroom for 40 min. The reduced and alkylated protein mixtures were precipitated by adding 6× volume of chilled acetone at −20 °C for 4 h. After centrifugation at 4 °C, 10,000g, 20 min, the subside was dissolved in 200µL 100mM TEAB (Applied Biosystems, Milan, Italy) and the protein was digested with Trypsin (Promega, Beijing, China) with a trypsin:protein =ratio of 1:50 at 37 °C for 12h, and equally divided into two equal parts. After trypsin digestion, peptides were dried by a rotary vacuum centrifugation (Christ RVC 2-25, Christ, Germany), redissolved in 0.4 M TEAB, and processed according to the manufacturer's manual for the 8-plex iTRAQ reagent (AB SCIEX, USA). Samples were labeled with the following iTRAQ tags as Sample SF, and Sample PF. The peptides were labeled with the isobaric tags and incubated at room temperature for 2 h. The labeled peptide mixtures were pooled and dried by Vacuum concentrator.

### LC/LC-MS/MS Analysis Based on Q-Exactive System.

Each fraction was resuspended with loading buffer [5 mM ammonium formate containing 2% acetonitrile (ACN); pH =10] and separated by high-pH reversed-phase liquid chromatography (RPLC, Acquity Ultra Performance LC; Waters, Milford, MA). The solvent A and solvent B is 2% ACN (pH=10, adjusted by ammonia) and 80% ACN (pH=10, adjusted by ammonia), respectively. The gradient elution was performed with 0% to 30% solvent B (2 to 38 min) and 30% to 100% solvent B (38 to 40 min) on a high-pH RPLC column (C18, 1.7 µm, 2.1 mm × 150mm; Waters, USA). LC-MS/MS analyses of all fractions collected were performed with a Nano Aquity UPLC system (Waters Corporation, Milford, MA) connected to a Q-Exactive hybrid quadrupole-Orbitrap mass spectrometer (Thermo, USA) equipped with an online nano-electrospray ion source. A 4-µL peptide sample was loaded onto the Thermo Scientific Acclaim PepMap C18 column (75 µm × 25 cm; 1.7-µm particle size) with a flow rate of 300 nL/min for 1 min and then separated on the analytical column (Acclaim PepMap C18, 75 µm × 25 cm) with a linear gradient, from 5% solvent B to 100% solvent B in 89 min, and then solvent B remained 100% in 6 min. The solvent A and solvent B used for separation is 2% ACN containing 0.1% formic acid and 80% ACN containing 0.1% formic acid, respectively. The column was re-

equilibrated in the initial condition for 30 min. The column flow rate was maintained at 300 nL/min, and the column temperature was maintained at 40 °C. An electrospray voltage of 1.9 kV was used.

The Q-Exactive mass spectrometer was operated in the data-dependent mode to switch automatically between MS and MS/MS acquisition. Survey full-scan MS spectra (m/z 350 to 1300) were acquired with a mass resolution of 70 K, followed by MS/MS scans with a resolution of 17.5 K.
